# AamA-mediated epigenetic control of genome-wide gene expression and phenotypic traits in *Acinetobacter baumannii* ATCC 17978

**DOI:** 10.1099/mgen.0.001093

**Published:** 2023-08-17

**Authors:** Jihye Yang, Yongjun Son, Mingyeong Kang, Woojun Park

**Affiliations:** ^1^​ Laboratory of Molecular Environmental Microbiology, Department of Environmental Science and Ecological Engineering, Korea University, Seoul, Republic of Korea

**Keywords:** antibiotic resistance, biofilm, DNA methylation, efflux pump, epigenetics, methyltransferase

## Abstract

Individual deletions of three genes encoding orphan DNA methyltransferases resulted in the occurrence of growth defect only in the *aamA* (encoding *Acinetobacter* Adenine Methylase A) mutant of *A. baumannii* strain ATCC 17978. Our single-molecule real-time sequencing-based methylome analysis revealed multiple AamA-mediated DNA methylation sites and proposed a potent census target motif (TTTRAATTYAAA). Loss of Dam led to modulation of genome-wide gene expression, and several Dam-target sites including the promoter region of the *trmD* operon (*rpsP, rimM, trmD,* and *rplS*) were identified through our methylome and transcriptome analyses. AamA methylation also appeared to control the expression of many genes linked to membrane functions (*lolAB*, *lpxO*), replication (*dnaA*) and protein synthesis (*trmD* operon) in the strain ATCC 17978. Interestingly, cellular resistance against several antibiotics and ethidium bromide through functions of efflux pumps diminished in the absence of the *aamA* gene, and the complementation of *aamA* gene restored the wild-type phenotypes. Other tested phenotypic traits such as outer-membrane vesicle production, biofilm formation and virulence were also affected in the *aamA* mutant. Collectively, our data indicated that epigenetic regulation through AamA-mediated DNA methylation of novel target sites mostly in the regulatory regions could contribute significantly to changes in multiple phenotypic traits in *A. baumannii* ATCC 17978.

## Data Summary

The authors confirm that the data supporting the findings of the present study are available within the article and/or its supplementary material. Transcriptomic data have been deposited in the NCBI database under Gene Expression Omnibus (GEO) accession GSE195621. Methylome data have been deposited in the NCBI database under Sequence Read Archive (SRA) accession SRR25365149 (Lab-WT) and SRR25365148 (*aamA* mutant).

**Impact Statement**

Although the emergence of multidrug pathogenic *A. baumannii* is a global health threat, and more than 17 000 genomes of this species have been deposited in the National Center for Biotechnology Information (NCBI) database at the time of writing this article, their genome-wide methylome analysis and AamA-recognition sites have been poorly explored. Our data revealed putative novel census AamA target motifs, which are densely positioned in 12 regions in the genome of *A. baumannii* strain ATCC 17978. Further methylome analyses and studies of AamA-controlled genes remain warranted because the size and structure of AamA proteins differ even among *A. baumannii* strains, and bacterial methylation patterns are known to vary under different conditions. Nevertheless, this study establishes a foundation for understanding the impact of AamA-mediated epigenetic control on the survival and virulence of *A. baumannii *.

## Introduction

Epigenetic regulation through DNA methylation has been recognized as a control mechanism of bacterial and archaeal gene expression, where methylation of specific DNA sites allows or hinders the binding of RNA polymerase or transcriptional regulators and changing chromosome superhelicity [1–3]. Bacteria harbour DNA-methylation-based restriction-modification (RM) systems (type I, II, and III) along with orphan methyltransferase (MTase) proteins lacking a restriction endonuclease partner, which are involved in cellular protection from foreign DNAs, DNA replication initiation, and DNA repair, or regulation of genes including those involved in virulence, motility and antibiotic resistance (AR) [4–7]. Unlike the type II RM systems that are composed of distinct MTase and restriction enzymes, type I and III RM systems appear to have a single multisubunit or complexes of multiple modification and restriction subunits, respectively [8]. Dam (a deoxyadenosine orphan MTase), one of the sequence-specific DNA methylases in *Escherichia coli *, encoded by the *dam* gene produces *N*-6-methyladenine (6mA), the most prevalent DNA modification in prokaryotes [9]. Dcm (a deoxycytosine orphan MTase)-mediated *N*-4-methylcytosine (4mC) and 5-methylcytosine (5mC) are less frequently found in genomes of Gamma-proteobacteria, including *Acinetobacter* species [8, 10]. The presence and number of orphan MTases differ in prokaryotic genomes, and most alpha-proteobacteria possess cell-cycle-regulated orphan MTase (CcrM), which methylates adenines found in different motifs (5′-GANTC-3′) and was initially discovered in cell-cycle regulation of *Caulobacter crescentus* [11–14]. CcrM (homologue YhdJ), which is also present in *E. coli* and *Salmonella enterica *, recognizes different consensus sequences (5′-ATGCAT-3′) [13, 14]. Although the genome sequences of *Acinetobacter* species revealed the presence of the *aamA* gene, its genomic target sites and AamA-mediated modulation of gene expression with consequent phenotypes have been poorly explored in *A. baumannii* strains [4].

Many methylation-dependent epigenetic switches can be explained by linking them to genome-wide methylation patterns (referred to as methylomes) in *Streptococcus pneumoniae, Campylobacter jejuni, Neisseria meningitidis* and *N. gonorrhoeae* [15–17]. These data and their interpretation have only recently become possible with the advent of single-molecule real-time sequencing (SMRT-seq) and nanopore-based sequencing techniques [8, 18, 19]. The SMRT-seq-based high-resolution mapping techniques can identify all types of methylated nucleotides (6mA, 4mC, 5mC and 5-hydroxymethylcytosine) in bacterial genomes at single-molecule resolution by measuring the time interval between the pulses of incorporation events, referred to as the inter-pulse duration (IPD), which reflects nucleotide modifications due to changes in the kinetics of the polymerase translocating the DNA template [8, 18, 20]. Many methylome studies revealed that many physiological and phenotypic variants in bacteria are regulated by epigenetic regulation. Modulating gene expression linked to the formation of extracellular matrix structures (e.g. *csgAD* gene) in the absence of *dam* methylation has been shown to result in reduced amounts of pellicle and biofilm formation along with changing colony morphology in *S. enterica* [21]. Types and amounts of bacterial surface polysaccharides are often governed by DNA methylation patterns, which leads to the production of opaque or transparent phenotypes (e.g. the promoters of *gtr* gene and the *agn43* together with *pap* genes in *S. enterica* and *E. coli *, respectively) [22, 23]. Differentially ectopic expression of multiple genes (e.g. *stdABC*, *filCD*, *sipC, cheR*) in the mutant of *dam* lacking pathogens including *S. enterica* and *Vibrio cholerae* leads to virulence attenuation according to lethality test using mouse models [24, 25].

Phase variation-dependent RM systems and orphan MTases alter global gene expression patterns, which also affect mutation rates, phenotypic heterogeneity and antibiotic resistance (AR) [5, 26]. AR in bacteria is made possible not only by the expression of intrinsic or horizontally transferred AR genes, but also by the methylation-mediated ON/OFF switches of genes involved in multiple pathways for bacterial survival against lethal antibiotic stress [26, 27]. Deletion of the *dam* gene makes *E. coli* cells susceptible under low doses of β-lactam and quinolone, however, adenine methylation patterns in wild-type *E. coli* appear to be very stable in the presence or absence of such antibiotics, which implies that the GATC methylome might guarantee stable structure of bacterial chromosomes for AR phenotype without having Dam-dependent differential gene expression under low doses of antibiotics [5]. Although molecular mechanisms of AR have been extensively studied, and multi-omics-based studies of AR have been performed in *A. baumannii* strains*,* Dam-dependent DNA methylation and consequent gene expression patterns have been poorly explored [28, 29]. In this study, deletion of the *aamA* (* Acinetobacter* Adenine Methylase A) gene and the SMRT-seq-based DNA methylome analysis was performed for the first time to investigate the genome-wide methylation patterns and direct or indirect linkages between the loss of AamA and phenotypic changes in *A. baumannii* ATCC 17978 under stress conditions.

## Methods

### Bacterial strains, culture conditions and scanning electron microscopy (SEM) analyses

The bacterial strains used in the present study are listed in Table S6. The *A. baumannii* ATCC 17978 (Lab-WT) used as the reference strain was provided by the American Type Culture Collection and maintained in our laboratory. The genome of the WT strain is available in the NCBI database (accession numbers CP000521). The *A. baumannii* strains included in the study were cultured at 37 °C in Luria–Bertani (LB) broth with constant shaking at 220 r.p.m. and aeration. All the strains were cultured overnight (O/N) for 16 h. Following this, each O/N cell culture was diluted 1/100 and additional incubation was performed to ensure that all the experiments were conducted in the mid-exponential phase (optical density, OD_600_, ~0.5). For SEM observation, cells (OD_600_, ~0.5) were incubated at 37 °C for 4 h and were first fixed with low-strength Karnovsky’s solution consisting of 2 % (v/v) paraformaldehyde, 2.5 % (v/v) glutaraldehyde and phosphate buffer (0.1 M) with final pH 7.2 for 4 h. Secondary fixation was done using 2 % (v/v) osmium tetroxide solution at 4 °C for 2 h. The fixed samples were gradually dehydrated with ethanol (30, 50, and 70 %) for 10 min each and placed on an aluminium stub O/N to dry at room temperature. These samples were then coated with platinum and assessed by FE-SEM analysis (Quanta 250 FEG; FEI, USA). Phenotypic observation of the WT strain and the *aamA* mutant using FE-SEM was conducted in triplicates.

### Phylogenetic analysis of AamA and protein alignment

The *aamA* and *dcmAB* genes encoding DNA methyltransferases in ATCC 17978 strain were annotated with the blast in NCBI database. To determine the conserved residues and functional domains of the AamA protein in the wild-type (WT) strain, we performed an alignment of the amino acid sequences of Dam from 28 other bacteria. These bacterial sequences exhibited a high similarity to the AamA protein sequence. The alignment was carried out using the ClustalW algorithm and the results were visualized using Mega-X software, version X. (http://www.megasoftware.net) with 1000 bootstrap replications. Phylogenetic trees were constructed based on the distance matrices using the maximum-likelihood method and the Tamura-Nei model. Tree distances were calculated using the branch-score distance with Treedist in phylip v3.695 [30, 31]. The protein sequences of Dam were visualized with Snapgene viewer version 6.0.5. The coloured blocks above indicate the sequence conservation, with the consensus thresholds of >70 % (orange) and >90 % (red), and below 50 % are coloured in sky blue (≤70 %) and blue (<40 %). The amino acids that match the consensus sequence were highlighted in yellow.

### CRISPR/cas9-based knockout of MTases and genetic manipulation of the *A. baumannii* lab-wt

The *aamA, dcmA* and *dcmB* gene-knockout mutants of the WT strain was constructed using the CRISPR-Cas9 system [32]. A pair of 20 bp spacer oligos and 80-nt oligos was designed to target the genomic locus and donor repair template for recombination using the RGEN tool (http://www.rgenome.net/) [33]. The 20 bp spacer oligonucleotides were phosphorylated and annealed to the pSGAb-km plasmid with a Golden Gate assembly reaction. Then, the plasmids were transformed into *E. coli* DH5α-electrocompetent cells, followed by plating onto an LB agar plate with KAN (50 µg ml^−1^) and incubation at 37 °C for 16 h. Successful cloning of the spacer was verified by PCR with the primers for each target gene’s spacer_F and the universal primer M13R (5′-CAGGAAACAGCTATGACC-3′) and by sequencing the target construct with the primer M13R. To induce the expression of the RecAb recombination system and Cas9 nuclease, isopropyl β-D-1-thiogalactopyranoside (IPTG, 1 M) was added to WT cells harbouring the pCasAb-apr plasmid. After incubating at 37°C for 2 h, the cells were briefly washed with distilled water and used to prepare electrocompetent cells, as described previously [32]. Then, the spacer-cloned pSGAb-km plasmid (200 ng) and 80-nt oligos (300 µM) were co-transfected into the IPTG-induced WT cells harbouring the pCasAb-apr plasmid by electroporation. Recombinant cells were plated onto an LB agar plate containing apramycin (100 µg ml^−1^) and KAN (50 µg ml^−1^) and then incubated at 37 °C overnight (O/N). Successful gene editing was verified by PCR amplification and sequencing of the target regions. For plasmid curing, freshly transferred cells were plated onto an LB agar plate containing 5 % (w/v) sucrose, incubated at 37 °C O/N, and then streaked onto LB agar plates without the antibiotics to confirm SacB counterselection.

To construct the *aamA* gene complemented strain, the broad-host-range vector pRK415 was digested with the restriction enzymes *Bam*HI and *S*acI. The fragments of the *aamA* were obtained by PCR amplification (1293 bp) and gel extraction and then inserted into the pRK415 vector via ligation. The fragment target gene-ligated pRK415 vectors were then used to transform into the *aamA* mutant strain by electroporation.

### SMRT-sequencing, motif discovery and transcription factor binding site prediction

Bacterial DNA methylation analysis was performed by SMRT (PacBio, USA) and high-throughput DNA *de novo* sequencing (DNAlink, Republic of Korea). Detection of base modification and methylated motif was conducted by employing the SMRT analysis portal following genome assembly. Briefly, genomic DNA samples extracted from mid-exponential phase (OD_600_, ~0.5) of the WT strain and *aamA* mutant were used for the sequencing and methylation analysis. Each genomic DNA sample was sheared using Megaruptor (ver 3.0) to collect 10 Kb DNA fragments, and then purified using the SMRTbell clean-up bead system to remove small fragments (<10 kb). Each purified 10 kb DNA fragment (500 ng) was used as the sequencing library using the SMRTbell Express Template Preparation kit (ver. 3.0). To reveal the genome-wide base modifications and the identification of 6mA, 4mC and 5mC residues in corresponding methylation (recognition) motifs, the SMRTbell library was sequenced on PacBio RS II System using SMRT Cell 8M (Pacific Biosciences) and Sequel Sequencing Kit 2.0 (Pacific Biosciences) on as previously described [34]. Reads were processed and mapped using BLASR mapper (ver. 12.0) and the Pacific Biosciences SMRT Analysis pipeline using the standard mapping protocol. Reads from the strains were mapped to Lab-WT reference whole genome sequence (accession numbers CP000521). The pulse width and inter-pulse duration (IPD) ratio for each base, which are altered when the DNA polymerase copies past a modified nucleotide, were measured, and modification for each base was determined using an in-silico control [35]. All methylome analyses using SMRT-sequencing were conducted in duplicate to validate methylation motifs in the WT genome. Condensed-6mA methylated regions based on SMRT-sequencing were classified into 12 regions on the genome. The promoter regions and the transcription factor and nucleoid-associated protein binding sites were predicted using web-based tools BPROM (http://www.softberry.com) and P2RP (http://www.p2rp.org) according to previous studies [36, 37].

### RNA-seq-based transcriptome analysis and reverse transcription followed by quantitative PCR (qRT-PCR)

Total RNA was extracted from mid-exponentially grown WT cells and *dam* mutant (OD_600_, ~ 0.5) using the RNeasy Mini Kit (Qiagen), according to the manufacturer’s instructions. The cells were incubated for 4 h in LB media (10 ml) at 37 °C. RNA sequencing was conducted on the Illumina NovaSeq 6000 platform (Illumina, USA) by DNA Link (Republic of Korea). The reference genome sequence was retrieved from the NCBI database (accession numbers CP000521). Relative transcript abundances are presented as fragments per kilobase of exon per million mapped sequence reads (FPKM). Genes exhibiting fold changes (FPKM values of *aamA* mutant cells/ FPKM values of control cells) greater than 2.0 and less than 0.5 were regarded as upregulated and downregulated genes, respectively. Only the genes with FPKM values greater than 50 in all transcriptome data were used in our gene expression analysis. All relevant RNA-seq data were deposited in the NCBI database under Gene Expression Omnibus (GEO) accession numbers GSE195621.

The qRT-PCR assay was conducted using the total RNA obtained from the WT strain and *aamA* mutant in the early-, mid-exponential and stationary phase (with OD_600_ values of ~0.4, ~0.5 and ~1.0, respectively) in LB media (5 ml) using the RNeasy Mini Kit (Qiagen, Germany). cDNA was synthesized from the total extracted RNA (3 µg each) and amplified using the primers (RT-qPCR primers) listed in Table S6. Relative expression levels of each gene were normalized to that of the 16S rDNA gene and analysed using the QuantStudio 3 Real-Time PCR instrument (Thermo Fisher Scientific, USA). All qRT-PCR procedures were conducted in triplicate from at least three independent cultures. Statistical analyses were performed using Student’s *t*-test (two-tailed) for comparisons of two groups (*P < 0.05, **P < 0.01, ***P < 0.001).

### Antibiotic susceptibility tests and EtBr efflux assay

Antibiotic susceptibility tests were performed using the broth dilution method on 96-well plates with antibiotics purchased from Sigma-Aldrich (USA). The following concentrations were tested: norfloxacin (NOR, 0.5–8 µg ml^−1^), meropenem (MEM, 0.25–8 µg ml^−1^), trimethoprim (TMP, 1–32 µg ml^−1^), rifampicin (RIF, 0.25–4 µg ml^−1^), polymyxin B (PMB, 0.5–8 µg ml^−1^), erythromycin (ERY, 0.5–32 µg ml^−1^), kanamycin (KAN, 0.5–32 µg ml^−1^), colistin (COL, 0.25–8 µg ml^−1^), azithromycin (AZI, 1–32 µg ml^−1^) and gentamicin (GEN, 0.5–8 µg ml^−1^). All isolates were cultured overnight in LB broth and then diluted (1 : 100) into fresh LB broth (5 ml) and incubated until they reached the mid-exponential phase (OD_600_, ~0.5). The cultured cells were transferred into a 96-well plate with each antibiotic, and the final cell numbers were adjusted (~10^6^ c.f.u. ml^−1^). Incubation was performed at 37 °C for 24 h to calculate the minimum inhibitory concentration (MIC). For the EtBr efflux assay, cells (OD_600_, 0.9–1.0) in potassium phosphate buffer (pH 7.0, 20 mM) containing 1 mM MgCl_2_ were incubated with CCCP (20 µM) and EtBr (10 µg ml^−1^) for 2 h at 30 °C. Cells were spun, washed and resuspended in fresh phosphate buffer (pH 7.0, 20 mM), and each well of 96-well plate were inoculated with 10^8^ c.f.u. of cells. The fluorescence signal of EtBr at excitation and emission wavelengths of 515 and 590 nm was monitored for the first 5 min, then efflux was activated by addition of 50 mM glucose, and the signal was monitored for 30 min. The EtBr signal was normalized by OD_600_. In order to confirm that EtBr did not compromise cell viability, cells were serially diluted and spotted on LB plates before and after the EtBr-treatment and incubated O/N at 37 °C. Both MIC test and EtBr efflux assay were conducted in triplicates.

### OMV quantification and measurement of biofilm formation

The lipid content of the OMV samples was indirectly quantified using the lipophilic dye FM4-64 (*N*-[3-triethylammoniumpropyl]−4-[4-(dibutylamino)styryl] pyridinium dibromide; Molecular Probes, Eugene, OR, USA and Life Technologies, Carlsbad, CA, USA). The lipophilic dye FM4-64, which intercalates into the outer membrane, is commonly used to stain and observe membranes of bacteria [38, 39]. To measure OMVs using this dye, cells were incubated for 16 h at 37 °C and 220 r.p.m. in 5 ml of LB broth. The cultured cells were harvested, and the supernatant was filtered through a 0.45-µm syringe filter. Aliquots of the filtrate (100 µl) containing OMVs were transferred into the wells of a dark 96-well plate. The FM4-64 dye was added to each well at a final concentration of 1 µg ml^−1^. The fluorescence (excitation and emission wavelengths of 515 and 610 nm) was measured using a fluorescence spectrophotometer (TECAN, Männedorf, Switzerland). To quantify biofilm production, mid-exponentially grown cells (OD_600_, ~0.5; 10^6^ c.f.u.) were inoculated in triplicates into LB broth (200 µl/well) on a 96-well plate. The volume of the cells was determined by converting the OD_600_ values of the cells incubated O/N. Uninoculated LB broth was used as the negative control. The microtiter plates were then incubated at 37 °C until mid-exponential (OD_600_, ~0.5) or stationary phases (OD_600_, ~1.0). After removing planktonic cells, the biofilm biomass was stained with crystal violet and solubilized with 95 % (v/v) ethanol, and its absorbance was measured at 595 nm. For confocal laser scanning microscopy (CLSM) observation of biofilm, the cells (OD_600_, ~0.5 or ~1.0) were stained with FilmTracerTM SYPRO Ruby (Invitrogen, USA) for 30 min at room temperature protected from light and then washed with distilled water. Confocal laser scanning microscopy (CLSM; Carl Zeiss, Germany) images were analysed and processed using the Zen 2.1 (Blue edition; Carl Zeiss, Germany) software. Quantification of OMVs formation and biofilm was conducted in triplicates.

### *Galleria mellonella* infection model

The *G. mellonella* larvae were obtained from SWORM (Republic of Korea). Healthy *G. mellonella* larvae weighing 200 mg were starved for 4 h at 20 °C before injection. Then, the larvae were placed on ice and injected via the last left proleg with 10 µl of cell culture (OD_600_, ~0.5 or ~1.0; 10^6^ c.f.u.) using 31-gauge, 6-mm-long needles (BD Ultra-Fine insulin syringes). Infected larvae were divided into two experimental groups with 20 larvae per group: (i) inactive control groups, which received phosphate-buffered saline (PBS, 10 µl) or cell cultures and (ii) bacterial treatment groups, which received either 1×10^6^ c.f.u. per larvae of ATCC 17978 or *∆aamA* cells. The larvae were incubated in a petri dish (90×15 mm, SPL Life Sciences) with 100 mg of wheat bran powder (MG Natural, Republic of Korea) at 37 °C in air for 96 h and inspected and scored every 12 h for death, failure to move in response to touch, and melanization.

### Colony expansion/motility tests

To determine the colony expansion phenotype, MTase (*aamA*, *dcmA*, and *dcmB*) deletion mutants and the Lab-WT strain were grown until the mid-exponential phase (OD_600_, ~0.5) in LB liquid. Each cell (2 µl, OD_600_, ~0.5) was placed in the centre of each LB agar (0.3 %, 0.5 %, and 1.5 % [w/v]) plate and incubated at 37 °C for 24 h. Colony expansion/motility images were observed using a Colony Doc-It Imaging station (Analytik Jena AG, Germany). Colony expansion/motility tests were conducted in triplicates.

## Results

### Identification and deletion analyses of three putative MTase genes

Genome and protein analyses revealed three putative genes encoding MTases in *A. baumannii* ATCC 17978 strain (referred to as WT): *aamA*, *dcmA* and *dcmB* (red blocks in Fig. S1A, available in the online version of this article). The annotated *aamA* and *dcmAB* genes exhibit 45.2%, 42.2%, and 51.5 % DNA identities with those in *E. coli* CV601. The Dam protein in *Campylobacter jejuni* NCTC 11168, with demonstrated in vitro activity, has 28.2 % protein identity with that of WT [40]. The three MTase genes are scattered throughout the genome, and their neighbouring genes, such as *holC* and *nrdR,* indicate that these regions (blue blocks in Fig. S1A) are possibly involved in DNA-replication and transcriptional regulation, although the functions of many other neighbouring genes (hypothetical; grey blocks in Fig. S1A) cannot be predicted [41, 42]. Consistent with other reports demonstrating that many MTase genes present in accessory regions of bacterial genomes are often associated with phage and DNA recombination genes [43, 44], our analysis showed incongruence between phylogenetic lineages and evolutionary relationships of MTase proteins (Fig. S1B; see Methods). Dimeric Dam proteins of *E. coli* CV601 are known to methylate DNA, but the multimeric status of other MTases is unclear, which might be attributed to differences in the size and consequently the number of domains in many MTases (Fig. S1B) [22, 45]. All MTases appear to possess two essential domains, N-terminal catalytic domain (the DPPY motif along with a cofactor binding FXGXG) motif and C-terminal DNA recognition domain, and several Dam proteins, including Dam in *E. coli* and *Neisseria gonorrhoeae,* have two copies of each catalytic and cofactor binding motifs (green and orange boxes, respectively, in Fig. S1B) [46]. However, the AamA protein in WT strain examined this study has a relatively shorter length with one copy of each important motif, indicating that Dam MTases have different degrees of shared relatedness and evolutionary history (red box in Fig. S1C).

**Fig. 1.**
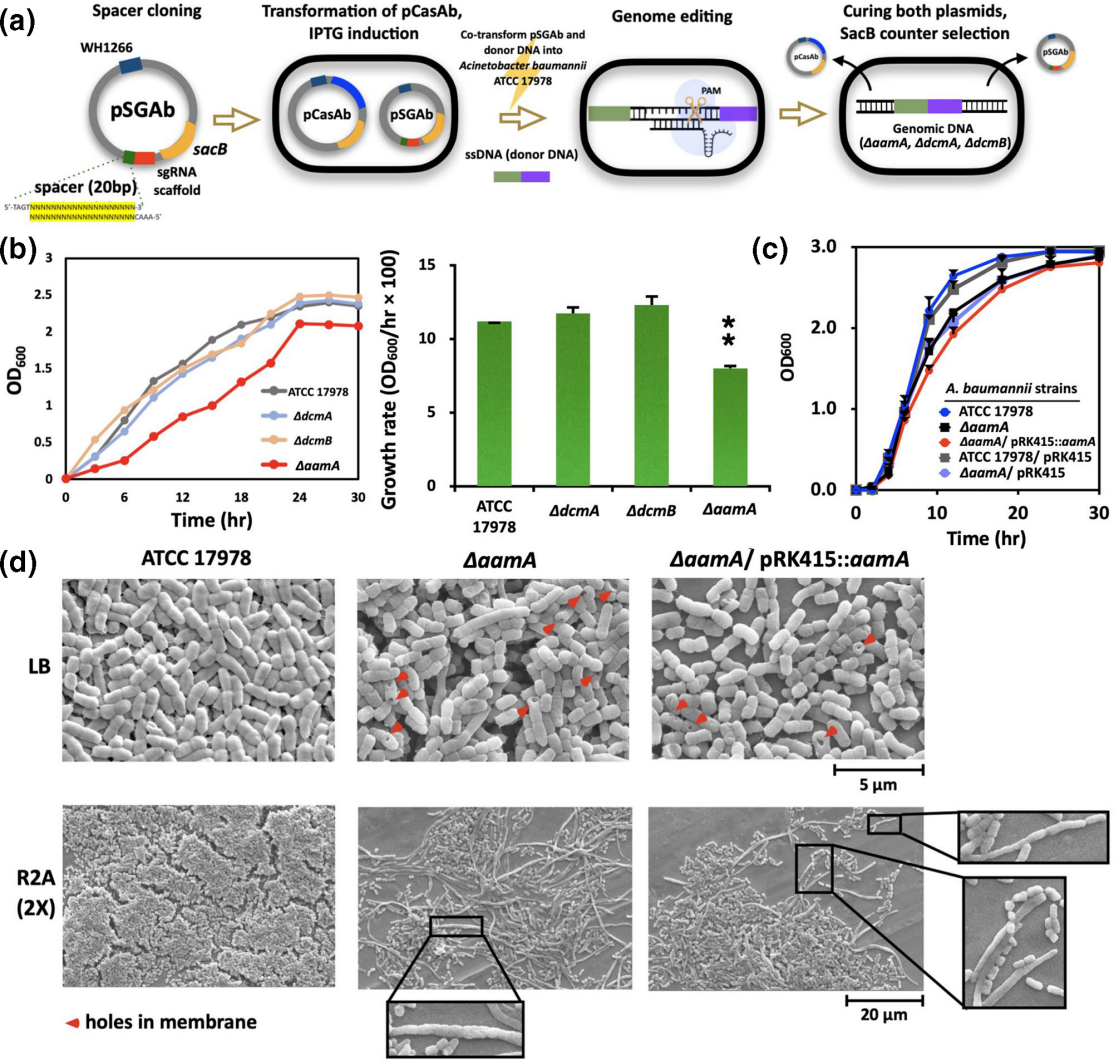
Knockout of AamA, DcmA and DcmB in WT strain. (**a**) Schematic overview of knockout of MTases in ATCC 17978 using CRISPR-Cas9 system. (**b**) Growth curve and rates (exponential phase) of each MTase deletion mutant and the control WT strain in 5 ml media. (**c**) Growth curve of *aamA* mutant and the complementary strain cultured in 50 ml media. All the *A. baumannii* strains were initially cultured at 37 °C in LB broth (pH 7) of various conditions with constant shaking at 220 r.p.m. and aeration. Following this, each overnight cultured cell population was diluted 1/100 and additional incubation was performed to ensure that all the experiments were conducted in the mid-exponential phase. Each medium was inoculated with 10^6^ c.f.u. ml^−1^ of each strain. (**d**) SEM images of mid-exponentially grown cells (OD_600_, ~0.5).

Individual deletions in all three MTase genes (*aamA*, *dcmA* and *dcmB*) were performed using the CRISPR/Cas9-based system through which a 200 bp fragment (starting from the first adenine nucleotide located between nucleotides 216–224) was successfully removed; in the case of the *aamA* gene, the cofactor binding FXGXG motif was completely deleted (see Methods) (Fig. 1a). Loss of AamA led to slower growth in *aamA* mutant ([8.0±0.2] / [*h*×100], *P<*0.05) compared to WT ([11.2±0.0] / [*h*×100]) in LB (5 ml), but no significant growth defects were observed in other *dcm* mutants (Fig. 1b) [47–49]. When growth media and conditions (aeration, pH) were changed to monitor the growth behaviour of each strain including the complementation strain (*∆aamA*/pRK415::*aamA*) and the vector controls (ATCC 17978/pRK415, *∆aamA*/pRK415), the growth retardation of the *aamA* mutant remained the same under all test conditions except for the R2A (2 x) broth media, and the *aamA* complemented strain failed to restore the growth rate (Figs 1c and S2). No plasmid-burden was confirmed by transforming the empty vector into the WT and *aamA* mutant strains (Fig. S2). Failure of our complementation assay and the absence of any growth defect in *aamA* mutant under certain conditions suggested that fine-tuning the expression of the *aamA* gene and AamA is necessary for the epigenetic control of target sites in the genome of *A. baumannii *. Scanning electron microscopic (SEM) image analyses showed no significant differences in the sizes of WT and the *aamA* mutant mid-exponentially grown in LB (OD_600_, ~0.5), but large holes were often detected on the surfaces of both the *aamA* mutant and the *aamA* complemented strains suggesting possible destruction of membrane structure (Fig. 1d). Interestingly, longer and filamentous cell morphologies were also observed in both the *aamA* mutant and the complemented strains under the R2A (2 x) broth condition, indicating strong linkage AamA protein abundance and surface structure of *A. baumannii* cells. Although the underlying molecular mechanisms of AamA-controlled membrane maintenance remain elusive, Aam protein deficiency suggested novel insights into cell surface modification (Fig. 1d).

### Detection of genome-wide methylation patterns in WT and the *aamA* mutant strains

**Fig. 2.**
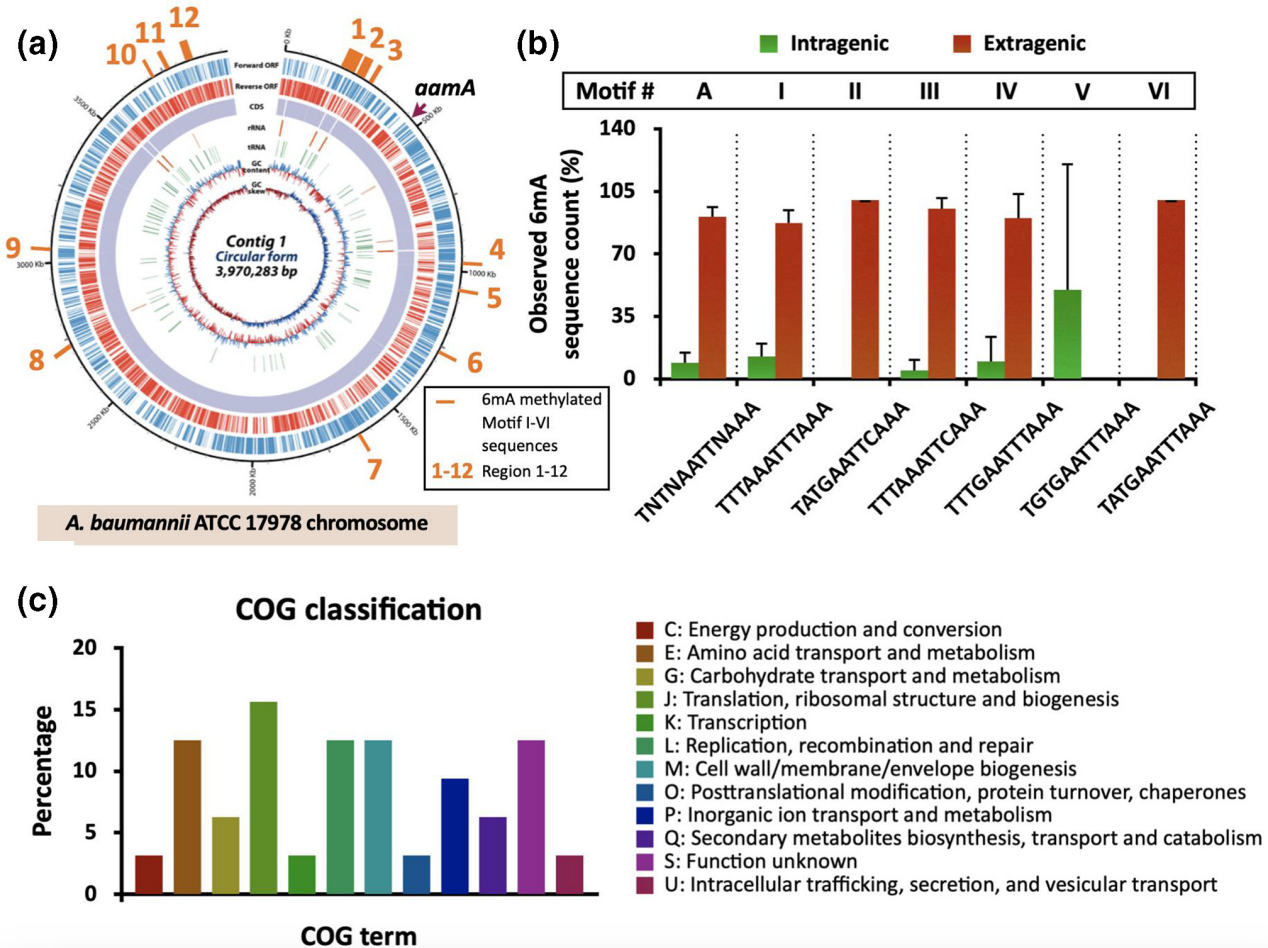
Methylome of the WT strain. (**a**) Circle map displaying the methylome of WT strain. The position of each methylation motif site in the genome of WT is indicated with an orange vertical bar. Each region is indicated above the motif positions. The position of the *aamA* gene is indicated with a purple arrow. (**b**) The observed sequence counts (%) of detected 6mA sites presented in the intragenic and extragenic regions of the WT chromosome. Full sequences of Motif A and I–VI are indicated in X-axis, which corresponds to Table 2. (**c**) Clusters of Orthologous Genes (COG) classification of detected methylation-site-associated genes and distribution of predicted protein functions of genes related to the unmethylated nucleotide sites in *aamA* knockout mutant.

To identify the methylation motifs in the WT genome, PacBio SMRT-seq methylome analyses were conducted in the WT and the *aamA* mutant strains (Fig. 2, see Data Set S1 for the complete lists of 6mA-type of adenine methylation). We performed SMRT-seq in replicate using Lab-WT and *aamA* mutant to confirm our methylome results. Although the SMRT technique can simultaneously detect all types of DNA methylation, and methylated cytosine residues detected in both strains (WT: 24,123[1st] and 701[2nd]; *aamA* mutant: 23,208[1st] and 711[2nd]) were more abundant than methylated adenine residues (WT: 2,259[1st] and 109[2nd]; *aamA* mutant: 2,062[1st] and 39[2nd]), our study focused on the 6mA-type of adenine methylation, because growth defect was only observed in the *aamA* mutant and 6mA was prevalent in all bacterial methylome analysis [8–14]. Since higher numbers of 6mA residues (first: 197 adenines; second: 70 adenines) were detected in WT, we speculated that these methylated adenines were parts of the DNA recognition sites directly or indirectly affected by AamA (Table 1). Only one type of methylation consensus motif (5′-TNTNAATTNAAA-3′; the methylated base is underlined, hereinafter referred to as Motif A), and a total of 59(1st) and 41(2nd) out of 281 Motif A-harbouring sites throughout the genome were deduced as possible AamA-modification sites accounting for 21.0 and 14.5 % of Motif A-like sequences, respectively. Our recognition motif A by AamA is atypical and longer compared to the previously reported methylation motifs (* C. crescentus *, 5′-GANTC-3′; *E. coli* and *S. enterica* 5′-ATGCAT-3′ [12–14]. Methylated adenines (6mA) on Motif A sequences were found on both the forward and reverse strands of WT genome with the highest mean inter-pulse duration ratios, which could not be observed in *aamA* mutant (IPD ratio, 3.6) (Table 2). A high percentage (first: 89.6 %, 59 out of 66; second: 62.1 %, 41 out of 66) of methylated adenines (6mA) on the subtype Motifs (Motif I-VI) was noticeable with the dominance of three Motifs (I, II, IV), which led to the proposal of a potent AamA target site with the sequence TTTRAATTYAAA in *A. baumannii* ATCC 17978 (Table 2). In the WT strain, the initial SMRT analyses uncovered a tenfold greater quantity of 6mA-type methylated adenine residues than the second SMRT analyses (Table 1). Nevertheless, the different amounts of 6mA detected in the replicated experiment and the presence of 6mA in the *aamA* mutant imply that slight alterations in the DNA superhelix and unidentified proteins might play a role in the methylation of additional N6-adenine sites during bacterial growth under the laboratory conditions (Table 1).

**Table 1.** DNA base modifications in ATCC 17978 (WT) and the *aamA* mutant. *N/A, not available

Strain	Modified base		Modification type	Motif string	No. detected		Mean lPD ratio		Mean coverage	
	first	second			first	second	first	second	first	second
* Acinetobacter baumannii* ATCC 17978	C (nDetected: 24,123)	C (nDetected: 701)	4mC	n/a	9886	0	1.5	1.6	381.1	436.4
			n/a		14 237					
	A (nDetected: 2,259)	A (nDetected: 109)	6mA	n/a	2200	68	3.6	3.2	411.2	644.2
				TNTNAATTNAAA (Motif A)	59	41				
* Acinetobacter baumannii* ATCC 17978, *∆aamA*	C (nDetected: 23,208)	C (nDetected: 711)	4mC	n/a	9612	0	1.5	1.6	380.4	439.5
			n/a		13 596					
	A (nDetected: 2,062)	A (nDetected: 39)	6mA	n/a	2062	39	1.5	1.6	381.3	644.3
				TNTNAATTNAAA (Motif A)	0	0				

**Table 2.** Adenine modifications (6mA) in ATCC 17978 (WT) and the *aamA* mutant. * N/A, not available

Strain		ATCC 17978						*∆aamA*				
**Motif**		**Chromosomal sequence count**	**6mA methylated sequence count**		**Methylation** **(%)**		**Region**	**Chromosomal sequence count**	6mA methylated sequence count*		**Methylation** (%)*	
			**First**	**Second**	**First**	**Second**			**First**	**Second**	**First**	**Second**
A	TNTNAATTNAAA	281	59	41	21.0	14.2		276	0		0	
I	TTTAAATTTAAA	14	14	11	100	78.6	1, 2, 4, 7, 12	14	0		0	
II	TATGAATTCAAA	1	1	1	100	100	1	1	0		0	
III	TTTAAATTCAAA	23	21	14	91.3	60.9	1, 2, 5, 8, 10, 11, 12	23	0		0	
IV	TTTGAATTTAAA	23	20	14	87.0	60.9	1, 2, 3, 8, 10, 11, 12	23	0		0	
V	TGTGAATTTAAA	2	1	0	50.0	0	9	2	0		0	
VI	TATGAATTTAAA	3	2	1	66.7	33.3	2, 6	3	0		0	
Total (Motif I-VI)		66	59	41	89.4	62.1		66	0		0	

AamA-recognized subtype Motif I-VI could be condensed into 12 regions on the genome as indicated in the circular genome map (Fig. 2a and Table S1). Among the total of 12 regions in our SMRT-sequence in replicate analyses, there were overlaps of five regions (regions 1, 2, 3, 4, and 6; a total of 60 methylated sites) having the same 44 methylated sites (73.3 %) (Table S1). The methylated sites were densely positioned at regions 1 and 2 (1st: 29 and 9 sites; second: 28 and 9 sites) and the IPD ratio of every single motif in these regions was higher (ranging from 3.5 to 6.1) than the average mean IPD ratio of detected motifs in regions 3–12 (1st: 2.6; second: 1.9) (Table S1). Region 12 also possesses multiple motifs (I, III, and IV) and a neighbouring gene (*smtA*) encoding a SAM-dependent-methyltransferase, suggesting possible hot spots for Aam-controlled gene expression. Motif A is dominantly present (first: 86.4 %; second: 95.1 %) in the extragenic regions, indicating that AamA protein methylates preferentially motif string in the promoter or regulatory regions (Fig. 2b). Exceptionally, motif V is only present (100 %) in the intragenic regions, which was not detected in the second methylome analyses.

Both methylation-site-associated genes and neighbouring genes near methylated regions were classified based on the COG (Cluster of Orthologous Groups of proteins) database (Fig. 2c and Table S2) [50]. The listed genes were assigned to 12 functional categories, with the top 5 COG classifications of translation, ribosomal structure, and biogenesis (J, 15.6 %), amino acid transport and metabolism (E, 12.5 %), replication, recombination and repair (L, 12.5 %), cell wall/membrane/envelope biogenesis (M, 12.5 %), and function unknown (S, 12.5 %) (Fig. 2c). The most abundant category (J) included the *rnpA* gene encoding an RNAse P protein (region 2) and four genes (*rpsP, rimM, trmD* and *rplS*) of the *trmD* operon involved in protein translation (region 10), indicating that AamA methylation could affect ribosomal stabilization and protein synthesis (Table S2) [51, 52]. Our methylome analysis also detected motif IV located upstream of *dnaA* gene linked to DNA replication (region 2), which is consistent with many other reports demonstrating the participation of 6mA on DNA replication initiation (Table 2) [53, 54]. Collectively, the 6mA patterns and positions described here forecast the engagement of AamA in many cellular processes such as ribosome stabilization/protein translation, DNA replication/repair, and membrane component biogenesis, which might be linked to the cellular adaptation of the ATCC 17978 strain under conditions tested in this study (Table S2) [55–57].

### Discoveries of differentially expressed genes and putative AamA-target sites

**Fig. 3.**
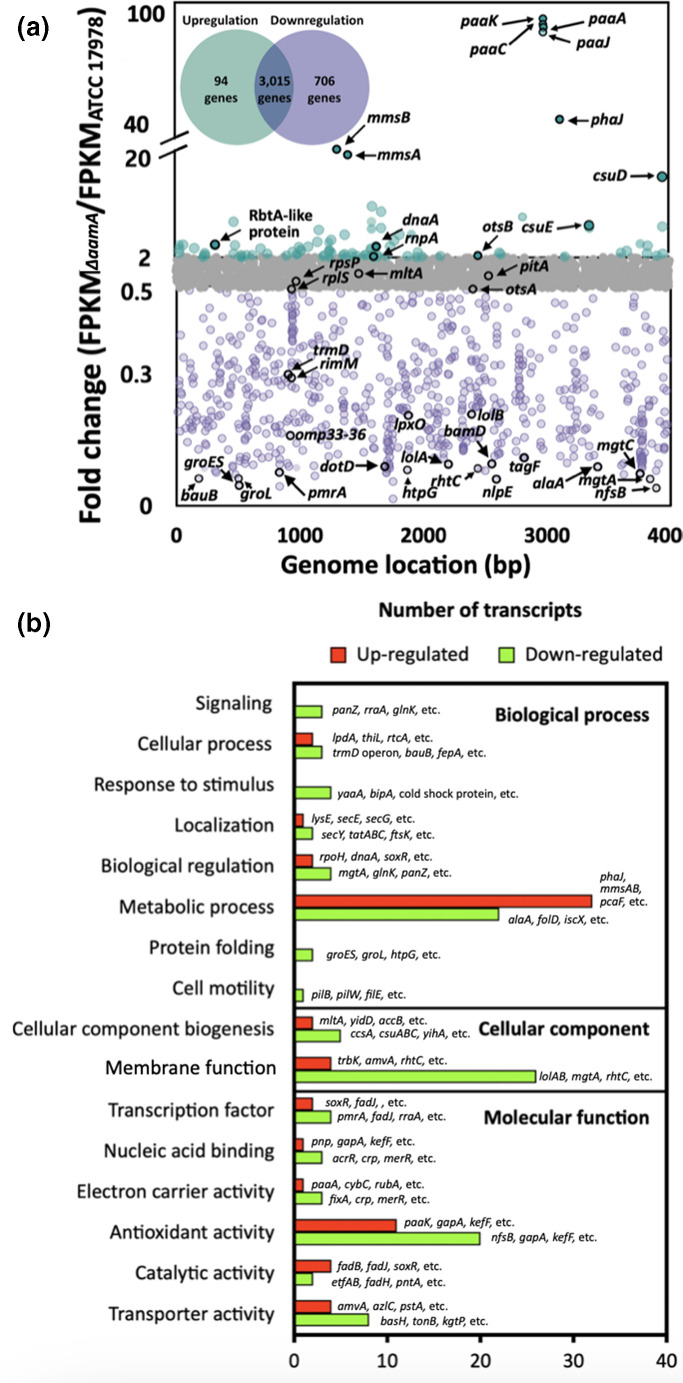
Transcriptome analysis and Gene Ontology (GO) classification of differentially expressed genes (DEGs). (**a**) Dot plots of DEGs between the *aamA* mutant and WT cells. The cells were incubated for 4 h in LB media (10 ml) at 37 °C. Genes exhibiting fold changes (FPKM values of *aamA* mutant cells/FPKM values of control cells) greater than 2.0 and less than 0.5 were regarded as upregulated and downregulated genes, respectively. In total, 94 and 706 genes were up- and downregulated, respectively, in *aamA* mutant in comparison with the Lab-WT. (**b**) The transcriptome GO IDs are mapped to GO classification. The terms of transcripts are assigned by the cellular function of respective upregulated (fold change ≤2.0) and downregulated (fold change >0.5) genes.

**Fig. 4.**
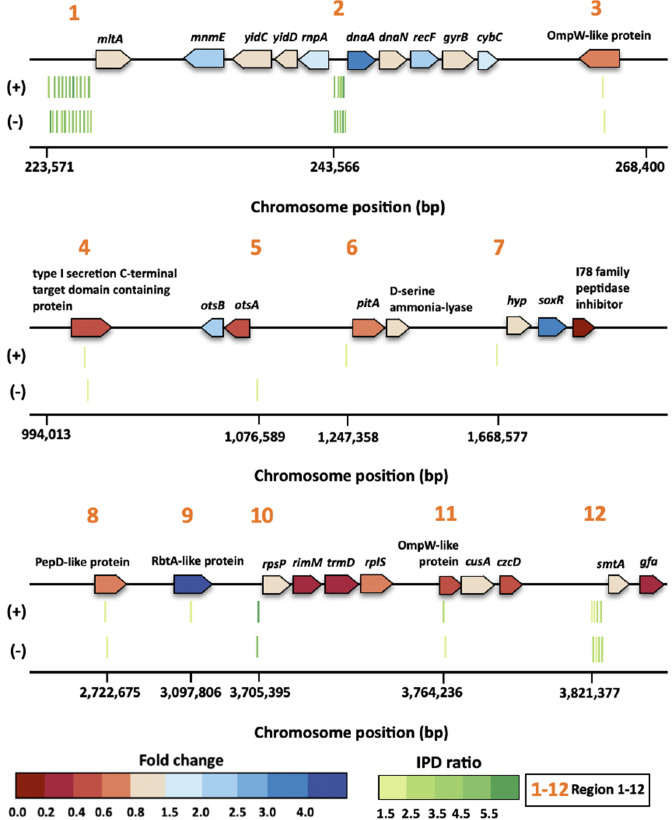
Expanded view of the region shown in Fig. 2a. Each arrow represents an individual gene to scale. The colors of the arrows represent relative degrees of differential gene expression (DEGs) of the *aamA* mutant cells versus WT cells based on the color scale at the bottom of the figure (fold change). The genomic organization of the different gene clusters was determined using databases for *A. baumannii* ATCC 17978 present in the MAGE platform. Vertical bars correspond to the distribution of the motifs, which were colored according to the color scale of the inter-pulse duration (IPD) ratio at the bottom (IPD ratio). The (+) and (–) signs correspond to the sense and antisense strands, respectively. Genomic locations according to the WT genome used as a reference are indicated below or within each gene (only the first are noted if they are correlative).

Transcriptomes of both WT and the *aamA* mutant strains were sequenced under the same conditions used for methylome analysis to understand AamA-mediated changes in genome-wide gene expression patterns (Fig. 3, see Data Set S2 for the complete lists of transcripts). Differentially expressed genes (DEGs; upregulated with FPKM fold change ≤2.0 or downregulated with FPKM fold change >0.5) showed that loss of AamA led to downregulation of 706 genes, including *trmD* operon, *lolAB*, and *lpxO*, while only 94 genes, including *paaK*, *mmsA*, and *csuD*, were upregulated among the total of 3815 genes (Fig. 3a and Table S3). There is no significant difference in the expression of the 3015 genes. The DEGs were classified into 16 biological functions using gene ontology enrichment analysis, revelling that most down-regulated genes were associated with membrane functions (e.g. *lolAB, mgtA* and *rhtC*), metabolic processes (e.g. *alaA, folD* and *iscX*), and antioxidant activity (e.g. *nfsB*, *gapA* and *kefF*), and the upregulated genes were mainly involved in other metabolic processes (e.g. *phaJ*, *mmsAB* and *pcaF*) (Fig. 3b). Interestingly, downregulation of genes involved in lipoprotein traffic (*lolAB*) and porin protein-folding (*bamAD*) pathways might affect the membrane stability and rigidity of AamA-mediated methylation (Fig. 3a and Table S3) [58–61]. The genetic organization of the *trmD* operon (*rpsP-rimM-trmD-rplS*) having motif IV (IPD ratio, 6.37[1st]) in the promoter region mirrors that in *E. coli *, and its expression was down-regulated (0.30–0.75-fold) in *aamA* mutant, partly supporting the defective growth phenotype in *aamA* mutant due to the linkage between this *trmD* operon and the 16S rRNA processing for translation (Figs 1 and 4) [62, 63]. It is worth noting that several genes having the intragenic N6-adenine methylation, including the *ompW-*like genes (region 3, 0.62-fold; region 11, 0.59-fold) and the *rbtA-*like gene (region 9, 6.16-fold), which are predicted to encode membrane proteins, were upregulated in the *aamA* mutant, although it is not clear how such modifications are linked to changes in gene expression (Fig. 3).

**Fig. 5.**
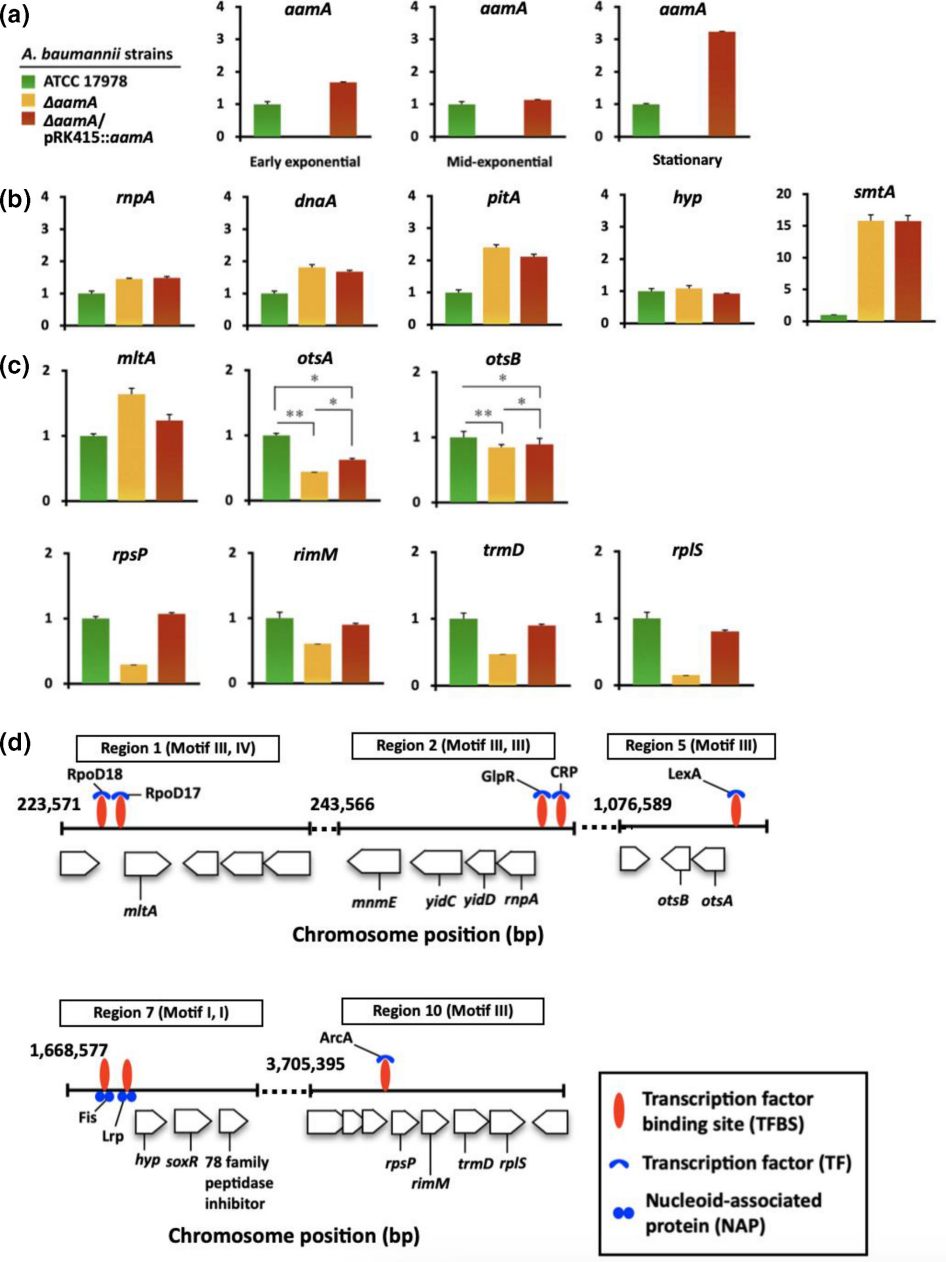
Relative gene expression analyses and transcription factor binding sites (TFBSs) overlapping with methylated sites. (**a**) The expression of the *aamA* gene in WT, *aamA* mutant, and the *aamA* gene-complemented strain in the early, mid-exponential, and stationary phase (OD_600_, ~0.4, ~0.5, and ~1.0, respectively). (b, c) The expression levels of the genes related to the methylated sites of the WT strain in the mid-exponential phase (OD_600_, ~0.5) of WT, *aamA* mutant, and the complementary strain. *, *P*<0.05; **, *P*<10^–2^. (**d**) Genetic regions overlap between the 6mA motif sites and transcription factor binding sites (TFBSs, red oval). The transcription factors (TFs) and nucleoid-associated proteins (NAPs) are shown in blue.

The expression levels of genes located downstream of predicted AamA-target sites were assessed by qRT-PCR in WT, the *aam* mutant, and the *aamA* complementation strains (Fig. 5). Growth-phase-dependent *aamA* expression has been reported in *E. coli *, and higher expression levels were observed at stationary phase (OD_600_, ~1.0; more than 3.2-fold) in the *aamA* complementation strain compared to WT (Fig. 5a) [64]. Thus, several possible target genes with AamA-mediated expression were monitored at the mid-exponential phase (OD_600_, ~0.5) to have the same levels of *aamA* expression in both WT and *aamA* complementation strains (Fig. 5a) [64]. In agreement with the transcriptomic data, the four tested genes (*rnpA, dnaA* and *pitA*) were upregulated (1.5–2.4-fold) when the *aamA* gene was deleted. Although the *aamA* gene was successfully complemented, and its gene expression levels were restored in the *aamA* complementation strain, the expression levels of the three target genes tested (*rnpA, dnaA* and *pitA*) did not reach those observed in WT, which is consistent with growth test results obtained with the same strains (Figs 5a, b and S2). However, our complementation assay showed that the expression levels of the *mltA* gene (1.6-fold)*, otsAB* operon (0.4–0.8-fold) and *trmD* operon (0.2–0.6-fold) in *aamA* mutant were successfully recovered to the levels observed in WT (Fig. 5c). The expression levels of several genes (*smtA*, *rpsP*, *rplS*) were not recovered in *aamA* complemented strain, because AamA-mediated on/off switch for gene expression might be attributed to the competitive target site binding of DNA-binding proteins (transcription factors [TFs] and nucleoid-associated proteins [NAPs]) at methylated or unmethylated sites rather than directly regulated genes [65, 66]. In total, eight methylation sites with motifs (I, III and IV) overlapping with the TF and NAP binding sites were identified using the web-based tools BPROM and P2RP (Fig. 5d). Interestingly, the presence of motif III in region 10 next to the *trmD* operon overlapping with a putative ArcA-binding site led us to hypothesize that the AamA-mediated methylation in motif III might decrease DNA-binding affinity of AcrA repressor and consequently boost the expression of the *trmD* operon, resulting in faster growth (Fig. 5d) [67, 68]. Further biochemical assays to test this speculation remain warranted. Our data suggested that the presence of many ON/OFF epigenetic switches possessing novel motifs in the regulatory sites could contribute to the fine-tuning of the expression of target genes, possibly by changing the DNA-binding affinities of TFs or NAPs.

### Alteration of membrane structure and phenotypic traits in the *aamA* mutant

**Fig. 6.**
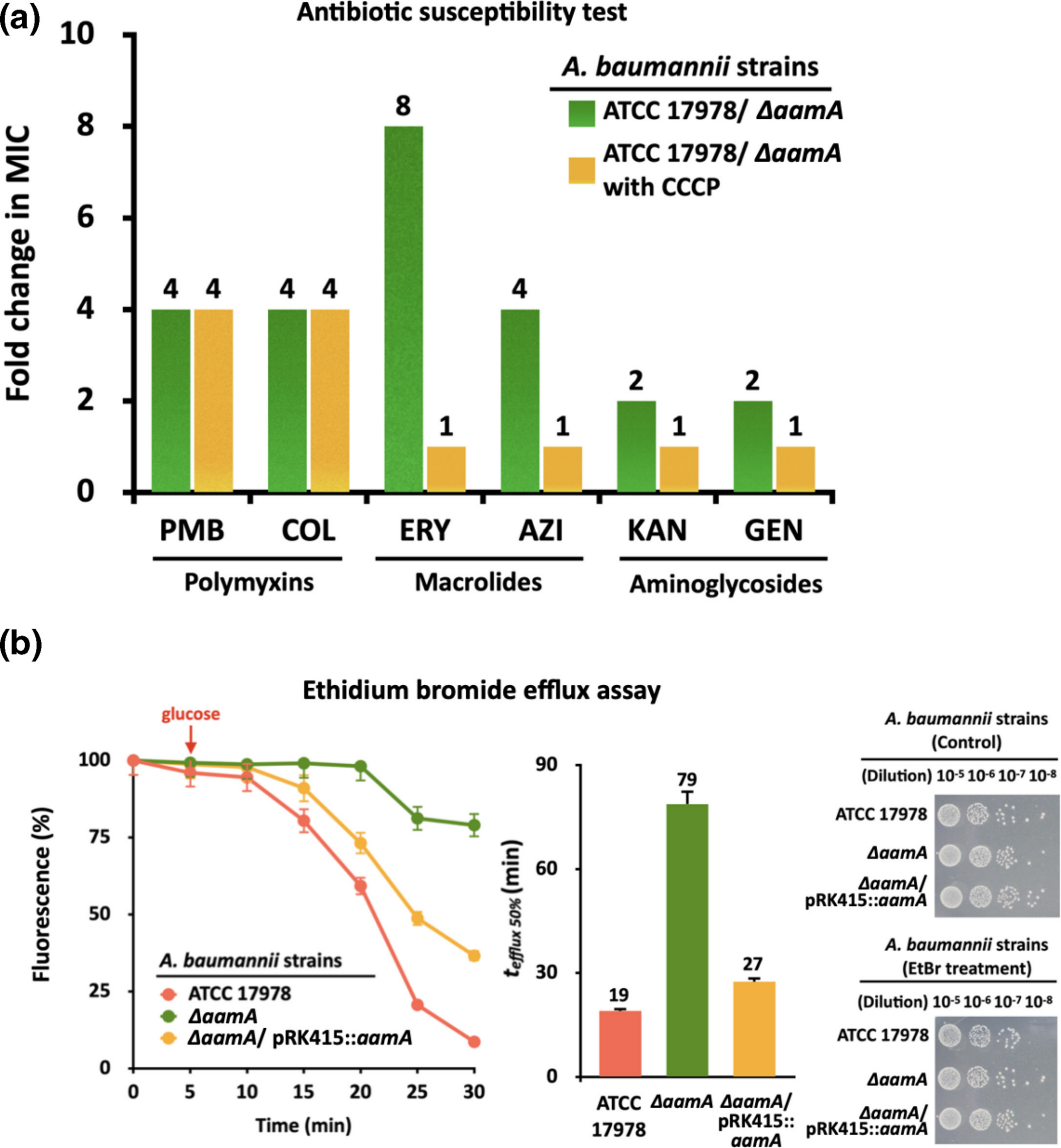
AamA-mediated multidrug resistance and efflux pump stability of the WT cells and *aamA* knockout mutants. (**a**) Fold changes in MICs from the antibiotic susceptibility tests for polymyxins, macrolides and aminoglycosides. The MICs of tested antibiotics are listed in Table S5. (**b**) Ethidium bromide (EtBr) efflux assay monitored for 30 min. Each strain was pretreated with EtBr for 2 h, and the addition of glucose boosted the activities of efflux pumps. The time (min) for each cell to reach 50 % EtBr is indicated in *t_efflux 50%_*. In order to confirm that EtBr did not compromise cell viability, cells were serially diluted and spotted on LB plates before and after the EtBr treatment (see Methods).

Aforementioned data showing the linkage between AamA and membrane integrity led us to investigate antibiotic susceptibility and membrane functions in WT and *aamA* mutant strains. Seven antibiotics of different classes, including polymyxin B (PMB), erythromycin (ERY) and kanamycin (KAN), were tested to determine the MIC for each strain. We found that loss of AamA made cells more susceptible to PMB, ERY, and KAN (fourfold, eightfold and twofold MIC reduction, respectively), and these decreases in MIC were recovered in the complementation strain (Fig. 6a and Table S4). When energy-driven efflux pumps localized in the plasma membrane are also responsible for AR [69, 70], cells exposed to carbonyl cyanide 3-chlorophenylhydrazone (CCCP), an efflux pump inhibitor, had the same levels of susceptibility to antibiotics (e.g. ERY, KAN) regardless of AamA, indicating that efflux systems in the membrane are partially affected by AamA activity. The same CCCP effect could not be seen in the presence of PMB acting on bacterial Lipid A (Fig. 6a and Table S5) [71]. Ethidium bromide (EtBr), a substrate of efflux pumps, was used to validate the direct or indirect association of AamA with efflux pumps. Glucose addition boosted the activities of efflux pumps so that EtBr-pretreated (2 h) WT cells, but not the *aamA* mutants, had faster gradual reduction of EtBr fluorescence (fourfold; 50 % EtBr loss [*t_efflux 50%_*]), and no EtBr-mediated cell death was observed during the assay (Fig. 6b, see Methods). The *aamA* complementation strain behaved similarly, but not to the same extent, with WT in the EtBr assay (Fig. 6b).

Further AamA-deficiency-associated phenotypic traits in relation to bacterial cell membrane surface, i.e. outer-membrane vesicles (OMVs), biofilm, virulence and colony expansion, were analysed (Fig. S3). Biogenesis of OMV appears to be coupled to the alteration of membrane integrity and affects bacterial AR [47, 72]. Several genes (e.g. *pal*, *bamD*, *ompA* family genes) known to be involved in OMV production through changes of membrane structures were downregulated in the *aamA* mutant, which might contribute to slightly enhanced OMV production (Fig. S3A). The OMV levels were diminished when the *aamA* gene was complemented in the mutant (Fig. S3A). However, increased biofilm formation, partly by the upregulated *csu* operon in the *aamA* mutant, could not be restored in the complementation strain probably due to the aforementioned reasons (Table S3, Fig. S3B). *Galleria mellonella* larvae-infection assay was performed to test the role of AamA on the virulence of the ATCC 17978 strain (Fig. S3C). Twenty larva per group were inoculated with 10^6^ c.f.u. of WT and *aamA* mutant strains (OD_600_, 0.5–1.0). Severe death of *G. mellonella* occurred within 4 days after infection with WT strain, but the groups infected with *aamA* mutant strain (both mid-exponential and stationary phase-grown cells) exhibited higher survival rates (Fig. S3C). No statistically significant difference in colony expansion was observed among the WT and mutant strains, although the *dcmB* mutant showed its swarming motility at the stationary phase (24 h at 37 °C) (Fig. S3). Although our data demonstrated *aamA*-mediated changes in virulence phenotypes and a possible role of *dcmB* for surface motility, underlying mechanisms still remain unclear and further experiments are needed to understand the genus *Acinetobacter *’s epigenetic control of many interesting phenotypic traits.

## Discussion

Although the evolutionary history of orphan AamA MTases is unclear in the genomes of *A. baumannii* strains, different sizes and sequence variations of *aamA* genes in *A. baumannii* strains might have resulted from horizontal gene transfer because of its different GC ratio (43 % vs. 39 % background GC ratio), which was partly supported by the presence of a *dam* gene with a different GC ratio in the accessory genome of *Pseudomonas aeruginosa* and the absence of *aamA* gene in some *Acinetobacter* species (the NCBI database) (Fig. S1C) [73]. Although a well-known Dam motif (5′-GATC-3′) has been extensively reported in *E. coli *, consensus target sites differ in bacterial species probably due to distinct structures of species-specific Dam proteins varying with multimeric status (e.g. 5′-GAAC-3′ in *S. enterica* and 5′-CGACTC-3′ in *C. crescentus *) [14, 74]. Recent SMRT-seq based methylome data from *C. jejuni* strains also identified multiple novel methylation sites and predicted several motifs (e.g. 5′-TAAYN_5_TGC-3′/5′-GCAN_5_RTTA-3′, 5′-GAGN_5_RTG-3′/5′-CAYN_5_CTC-3′, and more) without specification of Dam-recognition sites [75]. In the case of *A. junii* 65, an isolate from a water-rich environment and also a source strain for the production of type II restriction enzyme *Acc65I*, methylome analysis provided six methylation patterns without identifying Dam-specific sites [76]. Dam protein (also referred to as AamA, designated as A1S 0222 in the strain ATCC 17978) was experimentally shown to have *in vitro* functional activity using a DNA fragment with one *EcoR*I restriction site (5′-GAATTC-3′), and its methylation sites (5′-GAATTC-3′) were the proposed Dam recognition sites [4]. The potent census target motif we predicted (TTTRAATTYAAA) harbours several methylation patterns revealed by both the methylome (* A. junii* 65) and *in vitro* biochemical studies (AamA of the same ATCC 17978 strain) and can specify Dam recognition sites more precisely by expanding the DNA sequence length of the pattern (Table 2). However, further physiological and biochemical analyses are needed to develop a more comprehensive list of Dam target sites due to variations in methylation patterns under different conditions [77].

Although the linkage between hemimethylated states of some genes and DNA replication/cell cycle has been well-documented (e.g. SeqA and DnaA, CcrM), it is worth noting that faster bacterial growth conditions lead to more transient DNA hemimethylation during replication, which might stimulate or restrain the DNA-binding activities of many transcription factors (e.g. GcrA, CtrA, and PapI) and RNA polymerase [12, 78–80]. Dam protein may also affect the ON/OFF transcriptional switches of many genes (*mltA, rnpA, otsA, rpsP* and more) listed in our findings by methylating the target sites under certain conditions alone or in combination with other DNA binding proteins (CRP, LexA, Lrp, Fis, ArSMRT-seq-based; 5C, 5D and Table S3). Preferential binding of GcrA homologues to fully methylated target sequences has also been demonstrated in *Sinorhizobium meliloti* and *Brucella abortus *, suggesting a conserved GcrA–CcrM function in *Alphaproteobacteria* for controlling cell division, polarity, and motility [81]. In *P. aeruginosa* clinical strain TBCF10839, an orphan M.PaeTBCFII having 39 % amino acid identity to our AamA protein examined in our study recognized long and unusual methylation motif (5′-TRGANNNNNNTGC-3′), and the expression levels of many genes including *nosR* and *norB* of nitric oxide reductase are downregulated in *paeTBCFIIM* mutant strain [82].

Our data revealed that the *trmD* operon encoding four proteins for RNA modification (*trmD*, m^1^G37 of tRNA; *rimM*, 16S rRNA processing) and ribosomal proteins (*rpsP*, S16; *rplS*, L19) have promoter regions harbouring sites of Aam methylation and ArcA binding (Fig. 5d) [62]. Inactivation of the *trmD* gene decreases the synthesis of m^1^G37-tRNA, which prompts ribosomes to +1-frameshifts resulting in premature termination of protein synthesis, and consequent cellular growth retardations have been demonstrated in several bacterial species (e.g*. S. enterica* serovar Typhimurium, *E. coli* and *S. pneumoniae *) [52, 68, 83]. Loss of RimM also appears to inhibit the processing of 16S rRNA leading to a slowdown of protein translation and seven fold-reduction in the growth rate in *E. coli* [62, 84]. Interestingly, a recent TrmD study has also revealed that several membrane-associated genes (e.g. *acrB*, *lolB*, and *lpxD*) in gram-negative bacteria have multiple TrmD-recognized Pro (CCC[U]) codons, and the deletion of the *trmD* gene results in increased antibiotic susceptibility (e.g. KAN) due to defects in membrane lipoprotein and efflux pumps biosynthesis [85]. Consistent with these observations, our data demonstrated that the *aamA* mutant exhibited reduced expression of the *trmD* operon, reduction of antibiotic MICs (PMB, ERY and KAN), and lower activity of efflux pumps for EtBr (Fig. 5a and Table S4). Collateral damage and changes in bacterial behaviour such as OMV production, biofilm formation and *G. mellonella* infection might stem directly from AamA loss or indirectly through AamA-controlled genes including the *trmD* operon (Fig. S3). Collectively, our work provided for the first time comprehensive AamA-target sites and AamA-mediated changes in genome-wide gene expression, resulting in pleiotropic phenotypic changes in *A. baumannii* strain ATCC 17978.

## Supplementary Data

Supplementary material 1

Supplementary material 2
