# Supplementary material for: AamA-mediated epigenetic control of genome-wide gene expression and phenotypic traits in Acinetobacter baumannii ATCC 17978
Source: Microb Genom. 2023 Aug 17;9(8):mgen001093. doi: 10.1099/mgen.0.001093 (PMC10483419; doi:10.1099/mgen.0.001093)
Supplement: Supplementary material 1 [file mgen-9-1093-s001.pdf]

AamA-mediated epigenetic control of gene expression and phenotypic traits in *Acinetobacter baumannii* ATCC 17978

Jihye Yang[a], Yongjun Son[a], Mingyeong Kang, and Woojun Park*

Laboratory of Molecular Environmental Microbiology, Department of Environmental Science and Ecological Engineering, Korea University, Seoul, Republic of Korea

[a]These authors contributed equally to this work.

Running title: Adenine specific methylation in *A. baumannii*

Keywords: Epigenetics; *Acinetobacter baumannii*; DNA methylation; Antibiotic resistance; Efflux pump

[*]**Corresponding author:** Dr. Woojun Park, Department of Environmental Science and Ecological Engineering, Korea University, Seoul, Republic of Korea, 02841

E-mail: wpark@korea.ac.kr

Fax: +82-2-953-0737

Phone: +82-2-3290-3067

Table S1. Detected 6mA modified motif in the chromosome of WT strain. *(+), forward; (-), reverse strand; N/A, not available. Underlined for the methylated base.

Region 1	Position	Sequence (5′–3′)	Strand*	IPD ratio 1st	IPD ratio 2nd
1	223571	TTTAAATTTAAA	+	4.33	4.12
2	223572	TTTAAATTTAAA	-	6.05	5.63
3	223637	TATGAATTCAAA	-	4.41	N/A
4	223676	TTTAAATTCAAA	+	4.83	4.79
5	223677	TTTGAATTTAAA	-	4.76	4.36
6	223701	TTTGAATTTAAA	+	5.19	4.64
7	223702	TTTAAATTCAAA	-	4.44	4.51
8	223738	TTTAAATTCAAA	+	5.25	4.67
9	223739	TTTGAATTTAAA	-	4.67	4.74
10	223763	TTTAAATTCAAA	+	5.79	5.79
11	223764	TTTGAATTTAAA	-	4.02	4.26
12	223783	TTTAAATTTAAA	+	5.60	4.88
13	223784	TTTAAATTTAAA	-	5.06	3.81
14	223923	TTTAAATTTAAA	+	4.44	4.40
15	223924	TTTAAATTTAAA	-	5.39	5.16
16	223948	TTTGAATTTAAA	+	5.39	4.96
17	223949	TTTAAATTCAAA	-	4.47	4.65
18	223985	TTTAAATTCAAA	+	5.31	4.60
19	223986	TTTGAATTTAAA	-	5.06	4.63
20	224021	TTAAATTCAAA	+	6.00	5.60
21	224022	TTTGAATTTAAA	-	4.46	4.44
22	224057	TTTAAATTCAAA	+	4.38	4.02
23	224058	TTTGAATTTAAA	-	4.51	4.91
24	224094	TTTAAATTCAAA	+	4.98	4.66
25	224095	TTTGAATTTAAA	-	5.25	4.89
26	224119	TTTAAATTCAAA	+	3.93	4.03
27	224120	TTTGAATTTAAA	-	3.52	4.53
28	224182	TTTAAATTTAAA	+	4.78	4.66
29	224183	TTTAAATTTAAA	-	4.01	4.25
**Region 2**	**Position**	**Sequence (5′–3′)**	**Strand***	**IPD ratio 1st**	**IPD ratio 2nd**
30	243566	TTTGAATTTAAA	+	4.66	4.86

31	243567	TTTAAATTCAAA	-	4.58	4.29
32	243573	TATGAATTTAAA	-	5.22	4.66
33	243688	TTTAAATTTAAA	+	4.26	4.51
34	243689	TTTAAATTTAAA	-	3.70	4.47
35	243811	TTTGAATTTAAA	+	3.85	4.23
36	243812	TTTAAATTCAAA	-	6.02	5.54
37	243939	TTTGAATTTAAA	+	5.40	4.97
38	243940	TTTAAATTCAAA	-	4.49	4.36
**Region 3**	**Position**	**Sequence (5′–3′)**	**Strand***	**IPD ratio 1st**	**IPD ratio 2nd**
39	268346	TTTAAATTCAAA	+	2.34	2.06
40	268347	TTTGAATTTAAA	-	1.87	1.74
**Region 4**	**Position**	**Sequence (5′–3′)**	**Strand***	**IPD ratio 1st**	**IPD ratio 2nd**
41	994012	TTTAAATTTAAA	+	N/A	1.91
42	994013	TTTAAATTTAAA	-	1.62	1.72
**Region 5**	**Position**	**Sequence (5′–3′)**	**Strand***	**IPD ratio 1st**	**IPD ratio 2nd**
43	1076589	TTTAAATTCAAA	-	2.00	N/A
**Region 6**	**Position**	**Sequence (5′–3′)**	**Strand***	**IPD ratio 1st**	**IPD ratio 2nd**
44	1247358	TATGAATTTAAA	+	1.88	1.91
**Region 7**	**Position**	**Sequence (5′–3′)**	**Strand***	**IPD ratio 1st**	**IPD ratio 2nd**
45	1668577	TTTAAATTTAAA	+	1.79	N/A
**Region 8**	**Position**	**Sequence (5′–3′)**	**Strand***	**IPD ratio 1st**	**IPD ratio 2nd**
46	2722675	TTTGAATTTAAA	+	1.91	N/A
47	2722676	TTTAAATTCAAA	-	1.73	N/A
**Region 9**	**Position**	**Sequence (5′–3′)**	**Strand***	**IPD ratio 1st**	**IPD ratio 2nd**
48	3097806	TGTGAATTTAAA	+	1.73	N/A
**Region 10**	**Position**	**Sequence (5′–3′)**	**Strand***	**IPD ratio 1st**	**IPD ratio 2nd**
49	3705395	TTTGAATTTAAA	+	6.37	N/A
50	3705396	TTTAAATTCAAA	-	4.86	N/A
**Region 11**	**Position**	**Sequence (5′–3′)**	**Strand***	**IPD ratio 1st**	**IPD ratio 2nd**
51	3764236	TTTGAATTTAAA	+	2.80	N/A
52	3764237	TTTAAATTCAAA	-	2.34	N/A
**Region 12**	**Position**	**Sequence (5′–3′)**	**Strand***	**IPD ratio 1st**	**IPD ratio 2nd**

53	3821377	TTTGAATTTAAA	+	2.10	N/A
54	3821378	TTTAAATTCAAA	-	2.74	N/A
55	3821469	TTTAAATTCAAA	+	2.32	N/A
56	3821470	TTTGAATTTAAA	-	1.81	N/A
57	3821485	TTTAAATTTAAA	+	3.18	N/A
58	3821486	TTTAAATTTAAA	-	2.08	N/A
59	3821659	TTTGAATTTAAA	+	3.27	N/A
60	3821660	TTTAAATTCAAA	-	2.96	N/A

**Overlapped methylated region (1st and 2nd)**	**Overlapped methylated site (1st and 2nd)**	**Total methylated site (1st and 2nd)**
1, 2, 3, 4, 6	44 (73.3%)	60

Table S2. List of neighboring genes for which the motif detected in the intra/extra region.

Region	Gene symbol	Product	COG category
Translation, ribosomal structure and biogenesis			
2	*rnpA*	Ribonuclease P protein component	J
10	*rpsP*	30S ribosomal protein S16	J
	rimM	Ribosome maturation factor RimM	J
	trmD	tRNA (guanosine(37)-N1)-methyltransferase TrmD	J
	rplS	50S ribosomal protein L19	J
Amino acid transport and metabolism			
6	*metH*	Methionine synthase	E
	AUO97-RS12090	D-serine ammonia-lyase	E
7	*AUO97_RS14105*	Dihydrodipicolinate synthase family protein	E
8	*AUO97-RS00280*	Aminoacyl-histidine dipeptidase	E
Replication, recombination and repair			
2	*dnaA*	Chromosomal replication initiator protein DnaA	L
	AUO97-RS07330	DNA polymerase III subunit beta	L
	recF	DNA replication/repair protein RecF	L
	gyrB	DNA topoisomerase subunit B	L
Cell wall/membrane/envelope biogenesis			
1	*mltA*	Lytic murein transglycosylase	M
2	*yidD*	Membrane protein insertion efficiency factor YidD	M
3	*ompW*-like gene	TIGR04219 family outer membrane beta-barrel protein	M
11	*ompW*-like gene	Efflux RND transporter periplasmic adaptor subunit	M
Inorganic ion transport and metabolism			
6	*pitA*	Inorganic phosphate transporter family protein	P
11	*cusA*	CusA/CzcA family heavy metal efflux RND transporter	P
	AUO97-RS05085	Cation diffusion facilitator family transporter	P
Secondary metabolites biosynthesis, transport and catabolism			
4	*AUO97-RS10900*	Type I secretion C-terminal target domain containing protein	Q

12	*smtA*	Class I SAM-dependent methyltransferase	Q
Carbohydrate transport and metabolism			
5	*otsA*	Trehalose-6-phosphate synthase	G
	otsB	Trehalose-phosphatase	G
Post-translational modification, protein turnover, chaperones			
2	*mnmE*	tRNA modification GTPase MnmE	O
Intracellular trafficking, secretion, and vesicular transport			
2	*yidC*	Membrane protein insertase YidC	U
Transcription			
7	*soxR*	Redox-sensitive transcriptional activator SoxR	K
Energy production and conversion			
2	*cybC*	Cytochrome b562	C
Function unknown			
9	*rbtA*-like gene	CSLREA domain-containing protein	S
7	*AUO97-RS14095*	I78 family peptidase inhibitor	S
12	*gfA*	GFA family protein	S

Table S3. List of genes that were upregulated or downregulated in the *A. baumannii aamA* gene knockout mutant cells. Total RNAs were extracted from exponentially grown (OD_{600} ~ 0.5) WT cells at 37°C. Relative transcript abundances are presented as fragments per kilobase of exon per million mapped sequence reads (FPKM). Genes exhibiting fold changes (FPKM values of Δ*aamA* cells/FPKM values of WT cells) greater than 2.0 and lower than 0.5 were regarded as upregulated and downregulated genes, respectively. Genes with FPKM values below 50 in both dark control and blue light-irradiated samples were discarded. *C, cytosol; IM, inner membrane; P, periplasm; OM, outer membrane; E, extracellular components.

Gene symbol	Product	Subcellular localization*	FPKM		Fold change	Regulation
			Δ*aamA*	WT		
**Amino acid metabolic process**						
**Amino acid catabolic process**						
phaJ	Enoyl-CoA hydratase/isomerase family protein	C	114.106	2.32581	49.06	Up
mmsB	3-hydroxyisobutyrate dehydrogenase	C	52.1948	1.69429	30.81	Up
mmsA	CoA-acylating methylmalonate-semialdehyde dehydrogenase	C	82.9789	3.33757	24.86	Up
pcaF	3-oxoadipyl-CoA thiolase	C	80.0569	4.68901	17.07	Up
lpdA	Dihydrolipoyl dehydrogenase	C	94.6753	16.1978	5.84	Up
**Phenylacetate catabolic process**						
paaG	2-(1,2-epoxy-1,2-dihydrophenyl)acetyl-CoA isomerase	C	115.63	1.25	92.5	Up
paaK	Phenylacetate-CoA oxygenase/reductase subunit PaaK	C	137.536	1.50826	91.19	Up
paaC	Phenylacetate-CoA oxygenase subunit PaaC	C	110.892	2.13988	51.82	Up
paaA	1,2-phenylacetyl-CoA epoxidase subunit A	C	187.2	3.7	50.59	Up
paaJ	Phenylacetate-CoA oxygenase subunit PaaJ	C	125.734	2.64629	47.51	Up
paaZ	phenylacetic acid degradation bifunctional protein PaaZ	C	79.9059	3.17372	25.18	Up
paaF	Phenylacetate--CoA ligase	C	80.5972	6.94839	11.6	Up

**Amino acid biosynthetic process**						
alaA	pyridoxal phosphate-dependent aminotransferase	C	88.0837	1165.12	0.08	Down
folD	Methenyltetrahydrofolate cyclohydrolase FolD	C	27.2247	246.612	0.11	Down
proB	glutamate 5-kinase	C	22.4026	142.472	0.16	Down
**Oxidoreductase activity**						
nfsB	oxygen-insensitive NAD(P)H nitroreductase	C	11.8688	294.035	0.04	Down
gapA	glyceraldehyde-3-phosphate dehydrogenase	C	131.805	1660.11	0.08	Down
kefF	NAD(P)H-dependent oxidoreductase	C	14.3487	160.712	0.09	Down
etfA	FAD-binding protein	C	159.65	1762.27	0.09	Down
etfB	flavin reductase family protein	C	39.9863	367.11	0.11	Down
fadH	NADPH-dependent 2,4-dienoyl-CoA reductase	C	6.70986	54.6196	0.12	Down
pntA	NAD(P) transhydrogenase subunit alpha	C	93.2882	680.896	0.14	Down
dapB	4-hydroxy-tetrahydrodipicolinate reductase	C	38.8265	255.025	0.15	Down
AUO97-12525	NADH:flavin oxidoreductase/NADH oxidase family protein	C	21.5357	136.166	0.16	Down
rubA	rubredoxin	C	96.0047	559.18	0.17	Down
pcaC	Carboxymuconolactone decarboxylase family protein	IM	25.748	145.032	0.18	Down
**Other metabolic process**						
iscX	Fe-S cluster assembly protein IscX	C	39.9863	367.11	0.11	Down
ribA	GTP cyclohydrolase II	C	45.867	323.325	0.14	Down
kdpC	potassium-transporting ATPase subunit KdpC	IM	10.2461	59.6765	0.17	Down
phzF	PhzF family phenazine biosynthesis protein	C	8.95185	51.4908	0.17	Down

ycaC	hydrolase	IM	14.3062	72.3549	0.2	Down
**Biofilm formation**						
csuD	Csu fimbrial usher CsuD	O	120.5	7.2	16.64	Up
csuE	Csu fimbrial tip adhesin CsuE	E	190.4	25.1	7.59	Up
csuAB	Csu fimbrial major subunit CsuAB	E	3893.21	1481.62	2.63	Up
**Ferric uptake**						
bauB	siderophore-binding periplasmic lipoprotein BauB	P	10.8381	175.432	0.06	Down
fepA	TonB-dependent siderophore receptor	O	23.3991	246.119	0.1	Down
basH	Acinetobactin biosynthesis thioesterase BasH	O	8.02746	65.8185	0.12	Down
barB	acinetobactin export ABC transporter permease/ATP-binding subunit BarB	I	6.24184	50.6277	0.12	Down
AUO97-RS00825	TonB-dependent siderophore receptor	O	13.1953	102.782	0.13	Down
**Heavy metal resistance**						
nlpE	Copper resistance protein NlpE	O	155.604	2576.45	0.06	Down
arsC	Arsenate reductase	C	22.9947	206.762	0.11	Down
**Antibiotic resistance**						
pmrA	DNA-binding response regulator PmrA	C	9.77362	58.1146	0.17	Down
**Virulence**						
tagF	type VI secretion system-associated protein TagF	O	20.9224	144.71	0.14	Down
hicA	type II toxin-antitoxin system HicA family toxin	O	116.593	588.986	0.2	Down
**Protein folding**						
groES	co-chaperone GroES	P	487.351	10411.8	0.05	Down
groL	chaperonin GroEL	C	445.41	7170.59	0.06	Down
htpG	molecular chaperone HtpG	P	58.6006	723.315	0.08	Down
AUO97-RS03380	META domain-containing protein	C	27.5644	248.446	0.11	Down
**Cell wall/ membrane/ envelope biogenesis**						

mgtA	magnesium-translocating P-type ATPase	I	10.8066	177.972	0.06	Down
rhtC	Threonine efflux protein	I	40.7824	481.049	0.08	Down
kgtP	MFS transporter	I	21.1452	206.969	0.1	Down
AUO97-RS12380	SmpA/OmlA family protein	O	23.0418	193.953	0.11	Down
yiaD	OmpA family protein	O	23.0418	193.953	0.12	Down
mgtC	MgtC/SapB family protein	I	9.91621	76.5156	0.13	Down
dotG	DotG/IcmE/VirB10 family protein	O	58.5845	457.876	0.13	Down
pal	peptidoglycan-associated lipoprotein Pal	O	288.108	2271.13	0.13	Down
dotD	DotD/TraH family lipoprotein	O	95.6835	661.47	0.14	Down
AUO97-RS11615	OmpA family protein	O	1858.73	12053.8	0.15	Down
lolA	outer membrane lipoprotein carrier protein LolA	O	47.6368	308.664	0.15	Down
bamD	outer membrane protein assembly factor BamD	O	74.4344	482.126	0.15	Down
lytM	peptidoglycan DD-metalloendopeptidase family protein	I	40.1939	237.12	0.17	Down
motA	MotA/TolQ/ExbB proton channel family protein	I	108.121	631.603	0.17	Down
omp33-36	porin Omp33-36	O	2173.59	12209.9	0.18	Down
lpxO	lipid A hydroxylase LpxO	I	45.3199	219.952	0.21	Down
lolB	outer membrane lipoprotein LolB	O	24.5171	107.672	0.23	Down

Table S4. Antibiotic susceptibility of *A. baumannii* strains. * NOR, norfloxacin (Fluoroquinolone); MEM, meropenem (Carbapenem); TMP, trimethoprim (Sulfonamides-Trimethoprim); RIF, rifampicin (Rifampicin); PMB, polymyxin B (Polymyxin); ERY, erythromycin (Macrolides); KAN, kanamycin (Aminoglycosides).

**Strain**	**Antibiotics* (µg/ ml)**						
	**NOR**	**MEM**	**TMP**	**RIF**	**PMB**	**ERY**	**KAN**
ATCC 17978	2	1	32	1	2	8	4
Δ*aamA*	2	1	16	1	0.5	1	2
Δ*aamA*/ pRK415::*aamA*	2	1	16	1	2	8	4
ATCC 17978/ pRK415	2	1	32	1	2	8	4
Δ*aamA*/ pRK415	2	1	16	1	0.5	1	2

Table S5. Antibiotic susceptibility of WT (ATCC 17978) and Δ*aamA* strains with/without the efflux inhibitor CCCP. * PMB, polymyxin B; COL, Colistin; ERY, Erythromycin; AZI, Azithromycin; KAN, Kanamycin; GEN, Gentamicin.

Strain	Antibiotics (µg/ ml)					
	Polymyxins		Macrolides		Aminoglycosides	
	PMB	COL	ERY	AZI	KAN	GEN
ATCC 17978	2	2	8	16	4	1
ATCC 17978 (+ CCCP)	2	2	1	4	2	0.5
Δ*aamA*	0.5	0.5	1	4	2	0.5
Δ*aamA* (+ CCCP)	0.5	0.5	1	4	2	0.5
Δ*aamA*/ pRK415:: *dam*	2	2	8	16	4	1
Δ*aamA*/ pRK415:: *aamA* (+ CCCP)	2	2	1	4	2	0.5

Table S6. Bacterial strains, plasmids, and primers used in this study.

CRISPR/Cas9 primers	Sequence (5′–3′)	Reference
dcmA-spacer-F	TAGTGTTTGCTTGGCGATAAG	This study
dcmA-spacer-R	CTTATCGCCAAGCAACTCACCAAA	This study
dcmA-ssDNA (80nt)	AACCATGGCAAGGAATTGTTGACGTTATATCT GGCGGCTTCACGTTTTTCAGCATCTAACTTTG GAGCGCCCCATATCCG	This study
dcmA-sequencing-F	TTTGCTGAAAGGTGGTGCTTG	This study
dcmA-sequencing-R	CTCAAAGAAGAACGTCACAGGT	This study
dcmB-spacer-F	TAGTCGTCAATGCCTTTGGAAGGT	This study
dcmB-spacer-R	ACCTTCCAAAGGCATTGACGCAAA	This study
dcmB-ssDNA (80nt)	AGGCTAATCATCCCCATGCAAAGCATTATGT TCAAGATGTTAGGATGGGGACCTTTAATTGC AAAACGAGACAAAGCAAC	This study
dcmB-sequencing-F	GAGGCGGGATGAAGCTAAC	This study
dcmB-sequencing-R	TCCCACAATCCAGACAACGA	This study
aamA-spacer-F	TAGTAATTGCAACATGGCAAGCGC	This study
aamA-spacer-R	GCGCTTGCCATGTTGCAATTCAAA	This study
aamA-ssDNA (80nt)	GTGTGTCGATGCACCTGCTTTTAAAGAGTTGG GTGGCTATGTAATACCAGTGGTGTATTTAAAAG CTTCCACCAAGGGTG	This study
dam-sequencing-F	CACAAACGTCGTATGCAGC	This study
aamA-sequencing-R	ACCGCTCATCTGATTTGCTAGA	This study

qRT primers	Sequence (5′–3′)	Reference
qRT-16s-F	TGGTGCCTTCGGGAATCTAG	This study
qRT-16s-R	TGCGGGACTTAACCCAACAT	This study
qRT-aamA-F	CCTGCTGCTGAAATGTGGTG	This study
qRT-aamA-R	CTTTCGTATCTGGCGGTGGT	This study
qRT-rnpA-F	GCGCATGAAAGAAATCGAATAA	This study
qRT-rnpA-R	CCCAAATTTCGGCTGATGTAGA	This study
qRT-dnaA-F	CCTGAAATTGGAATGGCTTTTG	This study
qRT-dnaA-R	ACTTTTTCACAAGCATGCATCACT	This study
qRT-IPT-F	TGACGACCTAATGCCCAATG	This study
qRT-IPT-R	GACCCAATAGACAAGCTGCAATC	This study
qRT-hyp- F	TGCACGTTGCGATAGTATTTTACAT	This study
qRT- hyp- R	CAACCACATATCCAGAACATATTATAAACA	This study

qRT-SDM-F	ATGCACAAGCTCAAGGCAAAG	This study
qRT-SDM-R	ACATCCGGCTCGAAATATTGG	This study
qRT-mltA-F	TGGTGCCTTCGGGAATCTAG	This study
qRT-mltA-R	TGCGGGACTTAACCCAACAT	This study
qRT-otsA-F	TCCGGCAAAATTACTGTCGTT	This study
qRT-otsA-R	AGAAGCCCGTAGCCGTGAA	This study
qRT-otsB-F	GATTGCTGTAACAGGACGTGATG	This study
qRT- otsB-R	TGTAAGCCCGCTATAGGTAATTCA	This study
qRT-rpsP- F	CGTGCATTGCGGCTATCA	This study
qRT- rpsP- R	TGGCGGCGCAAAAAAA	This study
qRT-rimM-F	TGCGTTCTTCTGAATCAATGCT	This study
qRT- rimM-R	TGGAGCCAATGATGTGATGGT	This study
qRT-trmD-F	CAACGCAACCTTTTCAATCAAC	This study
qRT- trmD-R	TTTGCAGCGTTATCAACGAACT	This study
qRT- rplS-F	ACACCAACACCGCTAGAAATTTTAC	This study
qRT- rplS-R	AATCGCTAAGAAAAACCGTGGTT	This study
**Cloning primers**	**Sequence (5′–3′)**	**Reference**
aamA-pRK415-F (*B*amHI)	AAGGATCCATGAATTCAGAGCCTTCGGT	This study
aamA-pRK415-R (*S*acI)	AAGAGCTCCTTACCAAAGTGCGAGCTGTG	This study
