# Supplementary material for: AamA-mediated epigenetic control of genome-wide gene expression and phenotypic traits in Acinetobacter baumannii ATCC 17978
Source: Microb Genom. 2023 Aug 17;9(8):mgen001093. doi: 10.1099/mgen.0.001093 (PMC10483419; doi:10.1099/mgen.0.001093)
Supplement: Supplementary material 2 [file mgen-9-1093-s002.pdf]

AamA-mediated epigenetic control of gene expression and phenotypic traits in *Acinetobacter baumannii* ATCC 17978

Jihye Yang[a], Yongjun Son[a], Mingyeong Kang, and Woojun Park*

Laboratory of Molecular Environmental Microbiology, Department of Environmental Science and Ecological Engineering, Korea University, Seoul, Republic of Korea

[a]These authors contributed equally to this work.

Running title: Adenine specific methylation in *A. baumannii*

Keywords: Epigenetics; *Acinetobacter baumannii*; DNA methylation; Antibiotic resistance; Efflux pump

***Corresponding author:** Dr. Woojun Park, Department of Environmental Science and Ecological Engineering, Korea University, Seoul, Republic of Korea, 02841

E-mail: wpark@korea.ac.kr

Fax: +82-2-953-0737

Phone: +82-2-3290-3067

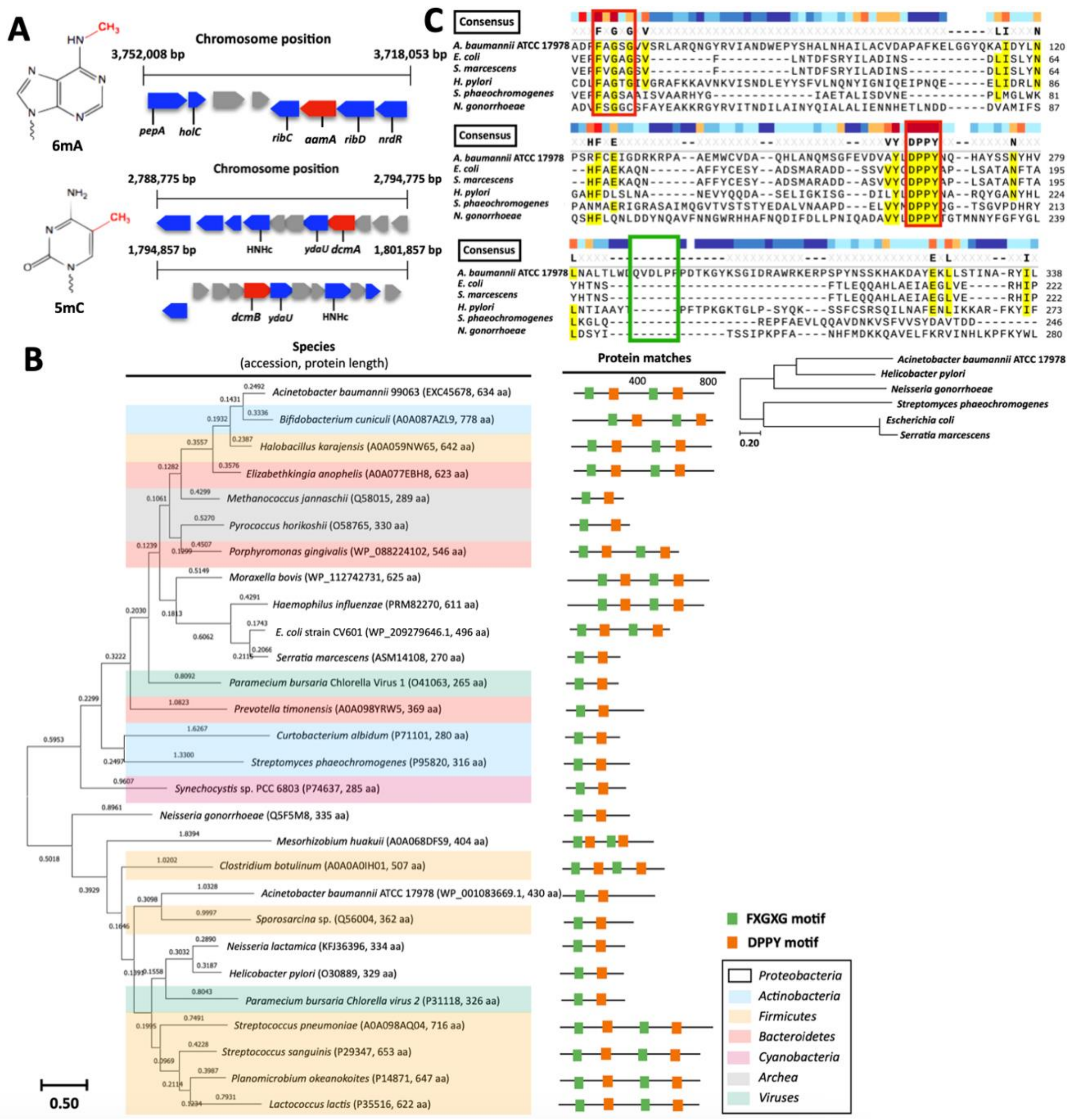

Fig. S1. Genomic context and phylogenetic analysis of MTases. (A) MTases in *A. baumannii* ATCC 17978. The respective targets (methylated DNA bases) of AamA, DcmA and DcmB are shown. (B) Phylogenetic tree of the Dam protein (n: 28) and AamA protein sequences of different organisms using MegaX. (C) AamA and Dam proteins alignment among *A. baumannii* (WP_001083669.1, 430 aa), *H. pylori* (O30889, 329 aa), *N. gonorrhoeae* (Q5F5M8, 335 aa), *S. phaeochromogenes* (P95820, 316 aa), *E. coli* (WP_209279646.1, 496 aa), and *S. marcescens* (ASM14108, 270 aa) using ClustalX. Conserved residues are highlighted in yellow. The phylogenetic tree obtained from the AamA and Dam alignment was indicated below.

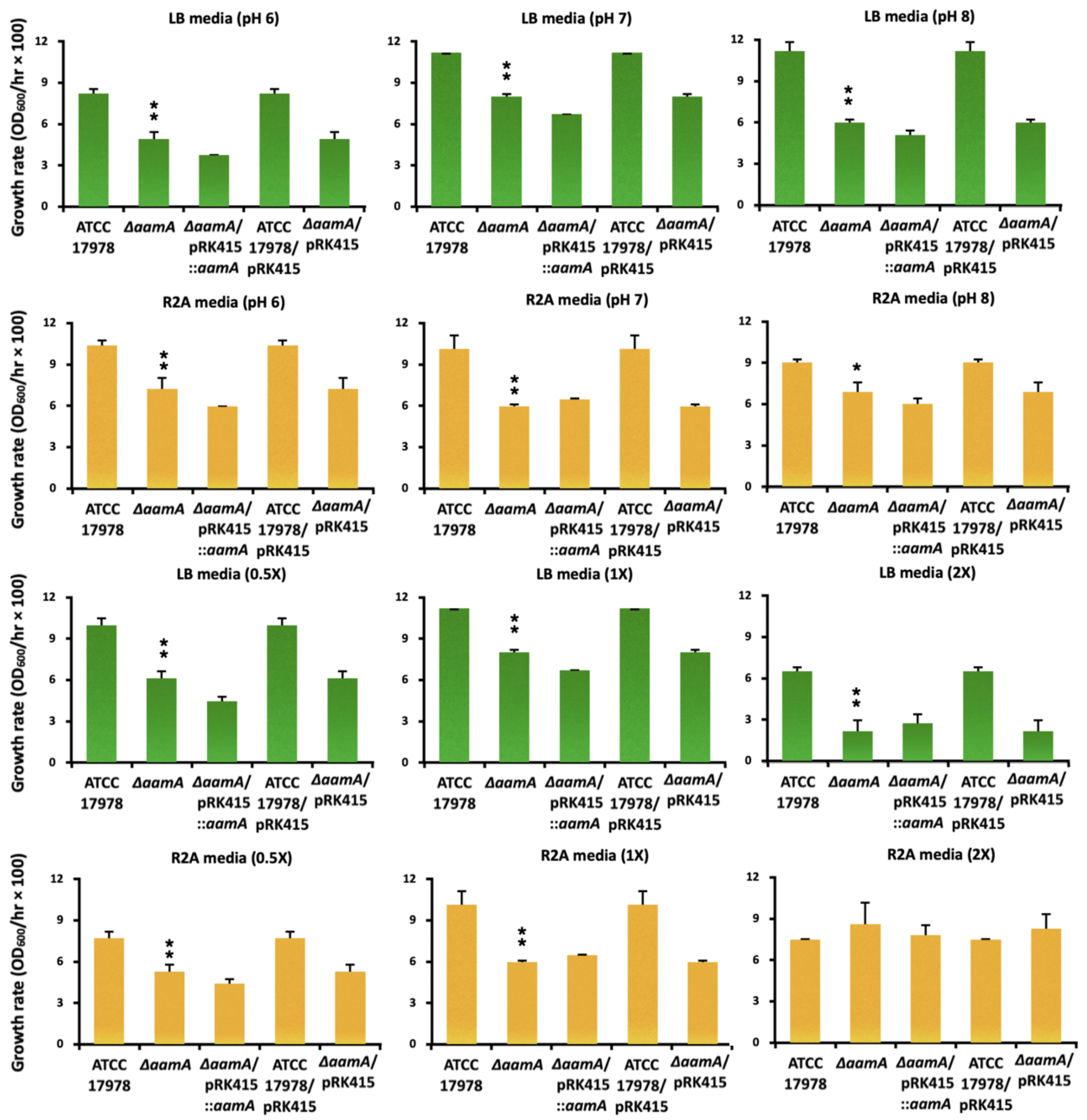

Fig. S2. Growth rates (exponential phase) of *A. baumannii* strains in various media. All the *A. baumannii* strains were initially cultured at 37 °C in LB or R2A broth of various conditions with constant shaking at 220 rpm and aeration. Following this, each overnight cultured cell was diluted 1/100 in each corresponding medium and additional incubation was performed to ensure that all the experiments were conducted in the mid-exponential phase (optical density, OD_{600} ~0.5). Each medium was inoculated with 10^6 CFU/mL of each strain. *$P < 0.05$, **$P < 10^{-2}$.

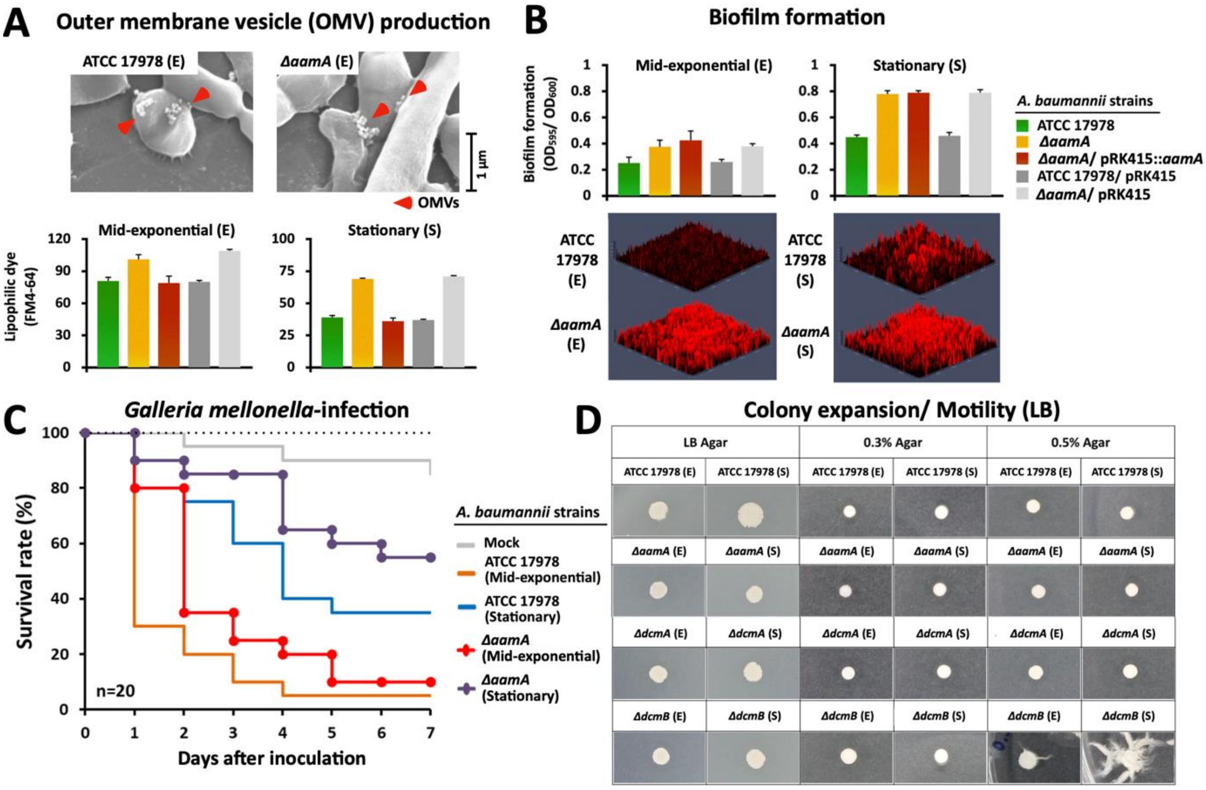

Fig. S3. Alteration of AR phenotypes in the *aamA* and *dcm* mutants. (A) Outer membrane vesicle (OMV) production observed by SEM and quantified by lipophilic dye (FM4-64). (B) Biofilm formation quantified by crystal violet and observed by confocal laser scanning microscopy (CLSM). (C) *In vivo*-infection test using *Galleria mellonella* larvae model. (D) Colony expansion/motility of MTase (AamA, DcmA, and DcmB) deletion mutants and the WT parent strain on agar plates. The overnight incubated cells were diluted 1/100 and additionally grown until mid-exponential phases of each strain (OD_{600} ~0.5). Each cell culture (2 µL) were placed in the center of the LB agar [1.5, 0.3, and 0.5% (w/v)] plate and incubated at 37 °C until mid-exponential (OD_{600} ~0.5) or stationary phase (OD_{600} ~1.0) of each corresponding strain.
